# Mitochondria‐Associated Endoplasmic Reticulum Membranes in Health and Diseases

**DOI:** 10.1002/mco2.70379

**Published:** 2025-09-18

**Authors:** Hangnan Hong, Zhenyang Guo, Junbo Ge, Hua Li

**Affiliations:** ^1^ Department of Cardiology Zhongshan Hospital, Fudan University Shanghai China; ^2^ Shanghai Institute of Cardiovascular Diseases Shanghai China; ^3^ State Key Laboratory of Cardiology Shanghai China; ^4^ National Clinical Research Center for Interventional Medicine Shanghai China; ^5^ Shanghai Clinical Research Center for Interventional Medicine Shanghai China; ^6^ Key Laboratory of Viral Heart Diseases National Health Commission Shanghai China; ^7^ Key Laboratory of Viral Heart Diseases Chinese Academy of Medical Sciences Shanghai China

**Keywords:** cancers, cardiovascular diseases, metabolic diseases, mitochondrial‐associated membranes, neurodegenerative diseases

## Abstract

Membrane contact sites enable organelles to interact closely, thereby coordinating cellular homeostasis and functional regulation. Among diverse subcellular membrane architectures, mitochondria‐associated endoplasmic reticulum membranes (MAMs) assume a crucial role in the physiological and pathological environments. A plethora of cellular processes are intertwined with MAMs, such as Ca^2+^ translocation, lipid metabolism, endoplasmic reticulum (ER) stress response, mitochondrial dynamics, and mitophagy. In the event of improper modulation of MAMs components, the incidence of diseases would surge remarkably. This review endeavors to expound upon the functions of key MAMs proteins in healthy state and decipher their regulatory mechanisms under physiological and pathological circumstances. In addition, we try to probe into the specific contribution of MAMs within the occurrence and development of diseases, and subsequently collate drug compounds and clinical trials that target MAMs components. Finally, we proffer our insights regarding the contentious perspectives and prospective research directions of MAMs. Understanding the roles and mechanisms of MAMs may potentially offer novel diagnostic biomarkers and treatment targets in clinical practice, paving the way for more precise and effective clinical interventions for common diseases.

## Graphical abstract

As pivotal regulators in diverse physiological and pathological processes, mitochondria‐associated endoplasmic reticulum membranes (MAMs) are critically involved in both health and diseases, including cancers, neurodegenerative disorders, metabolic diseases, cardiovascular diseases. Elucidating the functional mechanisms of MAMs may unveil novel therapeutic targets, facilitating the development of more precise and effective clinical interventions for various diseases.

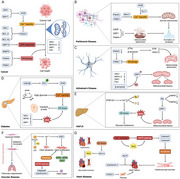

## 1 Introduction

Membrane contact sites (MCSs) are specialized regions where two organelle membranes closely appose, coordinating interorganelle functions and defined by specific protein compositions that stabilize these associations [1]. Among the various MCSs, the contact site between the endoplasmic reticulum (ER) and mitochondria, known as mitochondria‐associated ER membranes (MAMs), comprises portions of the ER and the outer mitochondrial membrane (OMM). These membranes remain closely apposed but do not fuse, allowing each organelle to retain its distinct properties [2]. MAMs were first discovered by electron micrographs in the early 1950s [3]. And in the early 1990s, MAMs were first biochemically isolated [4]. In 2009, the specific methods and procedures for isolating and purifying MAMs have been compiled into a well‐established protocol [5].

Despite the close apposition of their membranes, they do not merge and instead preserve their individual characteristics, endowing MAMs with a multiplicity of functions related to intracellular processes [6, 7, 8]. MAMs have been reported to assume a vital role in diverse cellular processes, encompassing Ca^2+^ transfer [9], lipid metabolism [10], ER stress and oxidative stress [11], as well as mitochondrial dynamics and mitophagy [12, 13]. Nowadays, emerging studies have uncovered potential links between health and MAMs. The regulated nature of these functions and processes is of vital significance in both human pathological and physiological contexts, presenting potential avenues as therapeutic targets.

In this review, we will initially direct our attention to the significance of MAMs within the membrane contact systems. We will meticulously elucidate the physiological functions regulated by MAMs and talk about its roles in health. Then, our focus will shift to investigating the intricate interplay that exists between MAMs and diseases. This section will examine how MAMs influence the development and progression of various diseases, as well as the potential mechanisms through which they might be targeted for therapy. And finally, we will offer a concise introduction to the drug compounds and clinical trials that target MAMs, and share our perspectives on the existing controversial issues surrounding MAMs. The comprehensive review will provide a holistic understanding of MAMs and their relevance in health and diseases, potentially providing a foundation for future research and clinical applications.

## 2 MAMs: Essential Biofunction Nexus Points

**FIGURE 1:**
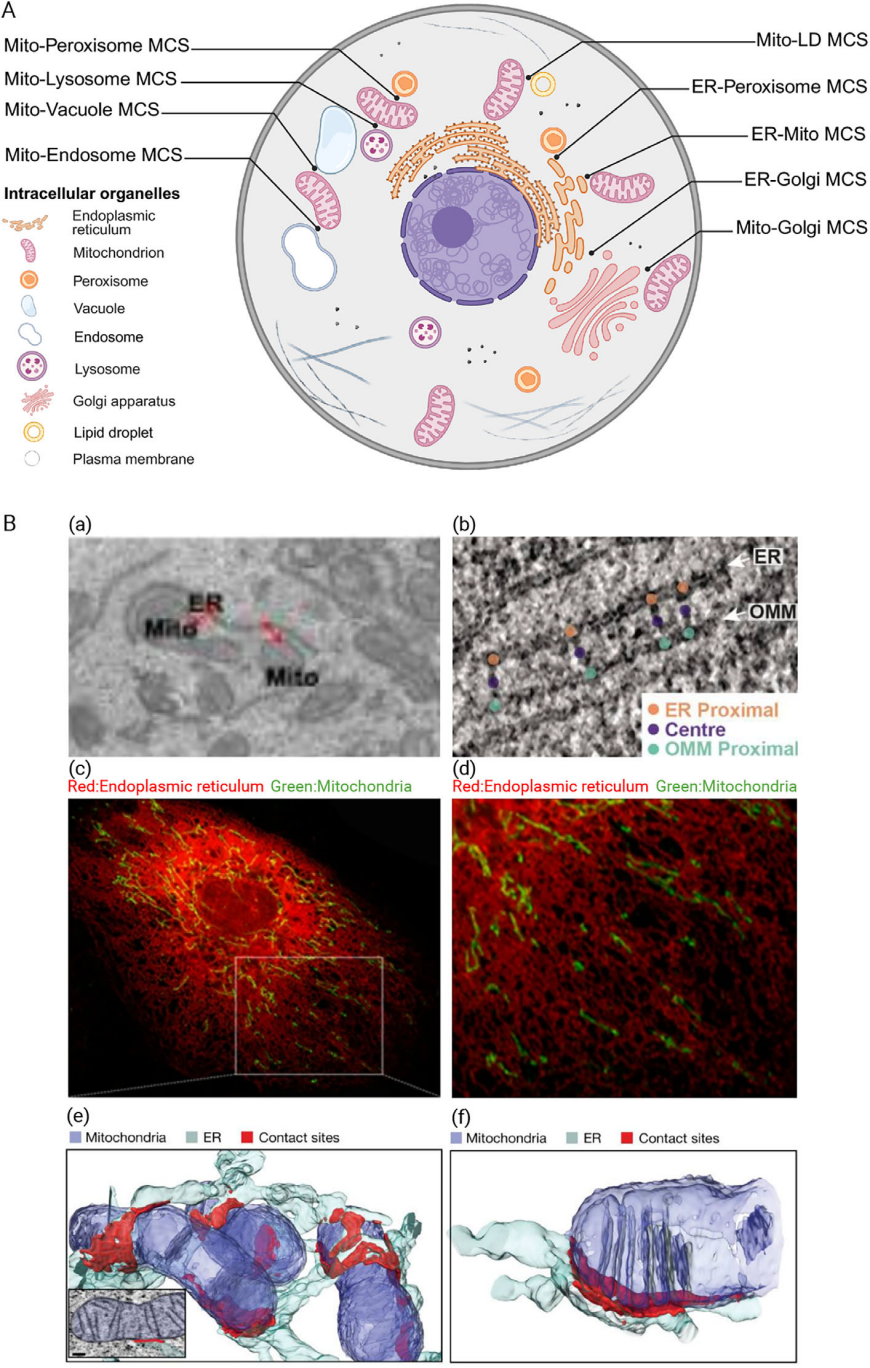
Overview of the membrane contact system. (A) Membrane contact sites between organelles exist commonly in mammalian cells. Organelles are not isolated entities. Their locations are organized by the cytoskeleton and membrane contact sites formed between different organelles. These membrane contacts serve as major regulatory hubs in the cytoplasm. In particular, the MCSs between the ER and mitochondria play a crucial role in regulating life activities (created in https://BioRender.com). (B) Representative images of MAMs. To observe the physical interaction between the ER and mitochondria in detail, different detections are used to observe it (created in https://BioRender.com). (a) Representative TEM image of ER–mitochondria contact [26] (adapted from Zhang, J. R. et al., 2024, *Nature Communication*, licensed under CC BY 4.0). (b) Representative image captured by cryoelectron microscopy. Coordinates of ER membrane anchor points (orange), center points (purple) and OMM anchor points (green) in electron cryotomograms [27] (adapted from Wozny, M. R. et al., 2023, *Nature*, licensed under CC BY 4.0). (c, d) Association between the ER (red) and mitochondria (green) was analyzed by confocal microscopy. Representative confocal images are displayed [28] (adapted from Wang, C. et al., 2021, *Nature Communication*, licensed under CC BY 4.0). (e) 3D focused ion beam‐scanning electron microscope (FIB‐SEM) reconstruction of the ER (cyan), mitochondria (blue), and ER–mitochondria membrane contact (red). Inset shows representative EM slice overlaid with segmentation masks [29] (adapted from Obara, C. J. et al., 2024, *Nature*, licensed under CC BY 4.0). (f) 3D reconstruction of the above membrane contact site with aligned cristae [29] (adapted from Obara, C. J. et al., 2024, *Nature*, licensed under CC BY 4.0).

MCSs are regions where two organelles are closely apposed, typically with a gap between 10 and 30 nm, serving as key platforms for interorganelle communication and signaling hubs [14, 15]. They are tethered by the protein–protein or protein–lipid interactions, which specifically modulate organelle functions [16]. A wide array of functions ranging from Ca^2+^ transfer, lipid metabolism, mitochondrial dynamics, mitophagy to cellular stress responses are mediated by different types of MCSs [17, 18, 19, 20]. MCSs are prevalently present in multiple organelles, and numerous contact sites have been identified hitherto. For example, the contact sites exist around the ER, such as ER–mitochondria, ER–plasma membrane (PM), ER–Golgi, ER–peroxisomes, and ER–lipid droplet (LD) contacts [21]. Concurrently, other contacts not involving the ER have also been unearthed: LD–peroxisomes, mitochondria–vacuole/endosome, mitochondria–lysosomes, mitochondria–PM, mitochondria–LD, mitochondria–peroxisomes, and so on [22] (Figure 1A). Among these contacts, the ER closely associates with 5%–20% of the mitochondrial surface, forming specialized ER–mitochondria contact regions known as MAMs [23]. MAMs exhibit a relatively stable nature and are of significant importance for maintaining internal environmental homeostasis including Ca^2+^ transfer, lipid metabolism, ER stress and reactive oxygen species (ROS) signaling, mitochondrial dynamics, and mitophagy [24]. To directly observe the structure of MAMs and further decipher their mysteries, transmission electron microscopy (TEM) was initially used in the 1950s [3]. Nowadays, with the advent and development of novel techniques like cryoelectron microscopy, immunofluorescence, and focused ion beam‐scanning electron microscopy (FIB‐SEM; Figure 1B), our understanding of the structure and functions of MAMs has been substantially deepened. As important signaling MCS. Recent studies have highlighted the pivotal role of MAMs in regulating diverse cellular processes. Dysregulation of MAMs has been linked to numerous pathological conditions, which are often marked by mitochondrial dysfunction [25]. Consequently, this prompts the recognition that it is imperative to conduct further in‐depth investigations into the interaction between MAMs and human body system.

**FIGURE 2:**
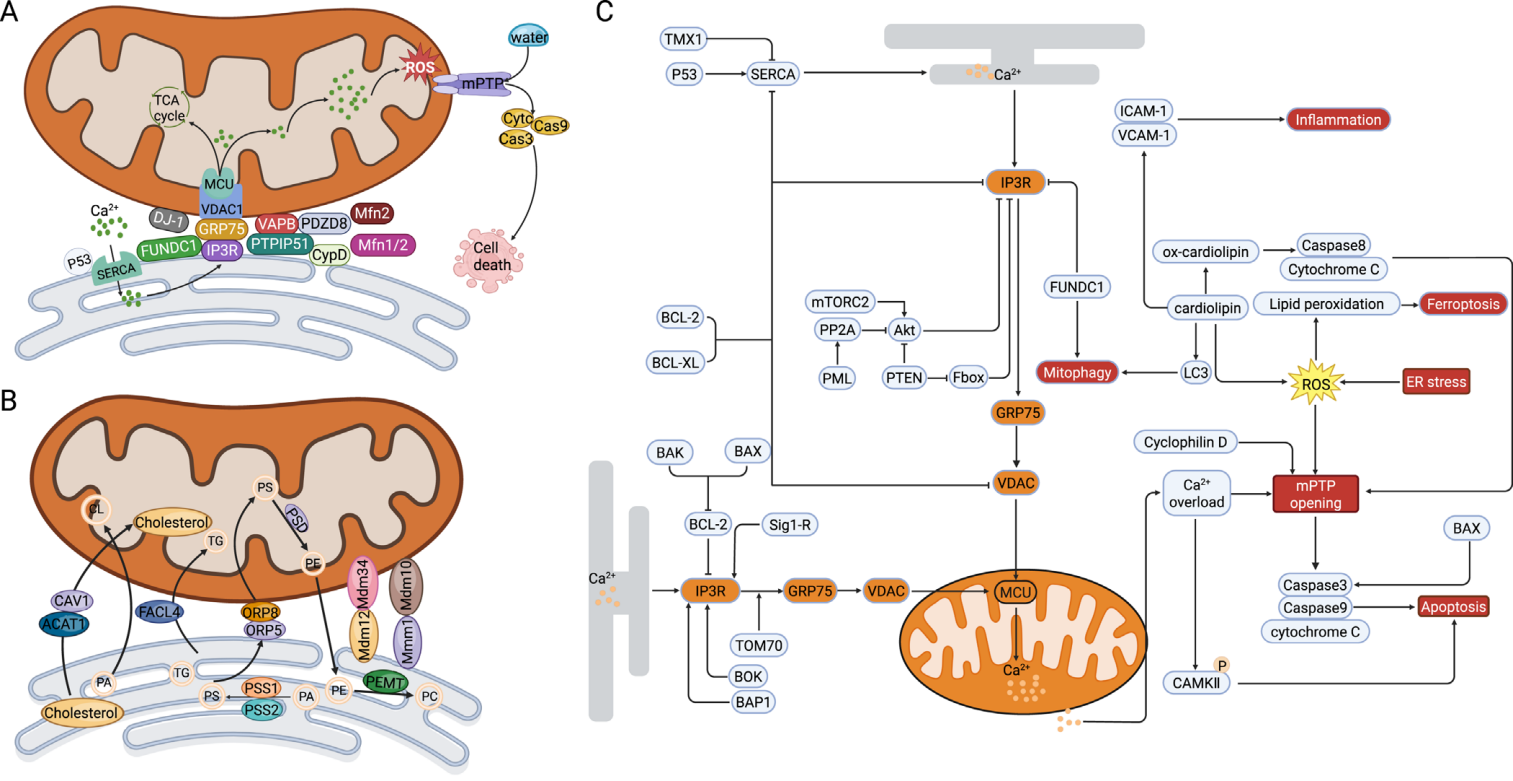
Molecular modulation of Ca^2+^ and lipid transfer. Graphic representation of the protein complexes which are components of the MAMs in the mammalian cells. Each part of them respectively fulfills different cellular functions and regulates distinct cellular processes (created in https://BioRender.com). (A) Components regulating Ca^2+^ transfer. When suffering from cellular stress, p53 binds to SERCA and modulates its redox status, resulting in increased Ca^2^⁺ loading into the ER. The accumulated Ca^2^⁺ is then transmitted to mitochondria via a tethering complex that includes IP3Rs on the ER, as well as GRP75 and VDAC1 on the mitochondrial surface. Ca^2+^ is then transported into the mitochondrial matrix via the MCU. Elevated mitochondrial Ca^2^⁺ levels stimulate TCA cycle enzymes, leading to increased NADH production and subsequently enhancing both ATP synthesis and ROS generation. Excessive mitochondrial Ca^2^⁺ uptake induces the opening of the mPTP, resulting in water influx and the release of cytochrome *c* into the cytosol. This event activates caspase‐9 and caspase‐3, thereby initiating the apoptotic cascade and ultimately leading to cell death. (B) Components regulating lipid metabolism. Phospholipid synthesis is topologically split between the ER and mitochondria. In the ER, PSS1, PSS2, and PEMT catalyze different steps converging at the synthesis of PS. PS is then transported into mitochondria by PSD for the conversion of PE. Mitochondrial PE can be traced back to MAMs and is converted into PC by PEMT. The transfer of PS to mitochondria also involves ORP5/8, MFN2, and CDS2. In addition, CAV1 has been proved to regulate ER–mitochondrial cholesterol transfer by binding to VDAC2. And ACAT1 catalyzes the conversion of free cholesterol to cholesterol esters between the ER and mitochondria. (C) Molecular network of Ca^2+^ transfer. MAMs play a crucial role in regulating Ca^2+^ transfer. IP3Rs–GRP75–VDACs is the major protein complex responsible for Ca^2+^ signaling between the ER and mitochondria. Mitochondrial Ca^2+^ uptake is indispensable for regulating cell fates. It is closely associated with diverse cellular processes like Krebs cycle and ATP production. However, excessive Ca^2+^ uptake will cause Ca^2+^ overload, thus leading to mPTP opening, which eventually results in cell death. In addition, other intracellular events are also associated with Ca^2+^ overload and mPTP opening such as ER stress and ROS production, which indicates that Ca^2+^ overload is a comprehensive process regulated by diverse factors.

## 3 Roles of MAMs in Health: From Functions to Mechanism

MAMs serve as a crucial nexus in maintaining normal cellular physiology, accommodating an expanding inventory of proteins that regulate essential biological activities. These include the regulation of Ca^2^⁺ transfer, lipid metabolism, ER stress responses, oxidative stress balance, mitochondrial dynamics, and mitophagy—all of which are fundamental for sustaining cellular homeostasis and overall health [30]. Beyond their tethering function, the diverse protein complexes within MAMs possess specialized roles that ensure the coordination of interorganelle communication under physiological conditions [8]. Notably, with a highly dynamic composition, MAMs contain more than 1000 proteins that participate in a broad spectrum of processes vital for normal cell function, while their dysregulation is increasingly linked to various diseases [31].

This section will provide a comprehensive summary of key proteins located at MAMs and their essential contribution to normal cellular physiology, as concisely summarized in Table 1. Then, to deepen our understanding of how MAMs take part in preserving cellular health under physiological conditions, we will examine the specific regulatory mechanisms that maintain interorganelle communication and cellular homeostasis.

**TABLE 1:** Key components and functions of MAMs.

Main roles	Related proteins	Regulatory functions	References
**Ca^2+^ transfer**	GRP75 VDACs IP3Rs	Acts as a connector between IP3R and VDAC1, mediating Ca^2^⁺ transfer from the ER to mitochondria. Regulator of Ca^2+^ influx and outflow of the mitochondrial membrane. Mediator of Ca^2+^ release from the ER into cytosol.	[32] [33] [34]
**Lipid metabolism**	ACAT1 ACSL4 Caveolin‐1	ACAT1 regulates cholesterol homeostasis by esterifying free cholesterol to cholesteryl esters, preserving their dynamic balance during resting conditions. ACSL4 catalyzes the synthesis of polyunsaturated fatty acid‐containing lipids, enhances lipid peroxidation, and supports cholesterol transport. Regulator of intracellular cholesterol transport and lipoprotein metabolism.	[35] [36] [37]
**ER stress**	IRE1α PERK	Activation of IRE1α governs ER stress and UPR. Activation of PERK governs ER stress and UPR.	[38] [38]
**Oxidative stress**	Ero1α P66Shc	Ero1α is linked to elevated ROS production and functions as a key regulator of oxidative stress. P66Shc has been reported to promote ROS production.	[39] [40]
**Mitochondrial dynamics**	MFN1 and MFN2 OPA1 DRP1	Participate in the fusion of outer mitochondrial membrane and tethering the ER and mitochondria. Mediator of the fusion of inner mitochondrial membrane. Involved in the initiation of mitochondrial fission.	[41] [42] [43]
**Mitophagy**	PINK1 Beclin1 FUNDC1	PINK1 enhances MAMs formation and promotes autophagosome precursor biogenesis. Facilitates ER–mitochondria tethering and enhances autophagosome formation. FUNDC1 interacts with DRP1 to trigger mitochondrial fission and initiate mitophagy.	[44] [44] [45]

### 3.1 Molecular Network of Ca^2+^ Regulation

The ER is the largest intracellular Ca^2+^ pool, releasing Ca^2+^ through inositol 1,4,5‐trisphosphate receptors (IP3Rs) and ryanodine receptors (RyRs) embedded in its membrane [46]. The ER forms an area of close contact with the OMM, enabling efficient mitochondrial Ca^2+^ transfer [18]. MAMs enable ER–mitochondrial Ca^2^⁺ exchange via multiple structural interfaces linking the two organelles [47]. Among the numerous MAMs‐associated proteins involved in Ca^2^⁺ transfer, IP3Rs–GRP75–VDACs is the principal protein complex [48] (Figure 2A). This complex is composed of ER‐resident IP3Rs, mitochondrial heat shock protein chaperone (GRP75) and mitochondrial voltage‐dependent anion‐selective channels (VDACs) [49]. Ca^2+^ is released from the ER via the IP3R channel and directed toward the VDACs in the OMM [50]. As a cytosolic chaperone, GRP75 bridges the IP3R and VDAC channels, promoting their interaction and improving the efficiency of mitochondrial Ca^2+^ uptake [49]. Unlike the highly Ca^2^⁺‐permeable OMM, which contains VDACs, the inner mitochondrial membrane (IMM) is impermeable, requiring specialized channels and transporters to mediate Ca^2^⁺ uptake. The mitochondrial calcium uniporter (MCU) protein, located in the IMM, facilitates Ca^2+^ entry into the mitochondrial matrix and interacts closely with the OMM protein VDAC1 [51, 52]. Once Ca^2+^ traverses through the OMM, it can use the MCU to translocate across the IMM into the mitochondrial matrix [53]. Molecular partners like cyclophilin D (CypD), a Ca^2^⁺‐sensitive mitochondrial chaperone foldase, also interact with the IP3Rs–GRP75–VDACs, regulating ER–mitochondria crosstalk and transporting Ca^2+^ from the ER to mitochondria [54]. Besides the IP3Rs–GRP75–VDACs complex, a few other proteins are also implicated in Ca^2+^ translocation through their interaction with IP3Rs. For example, a constitutively active form of glycogen synthase kinase 3β (GSK3β) has been shown to localize at MAMs as part of the IP3Rs–GRP75–VDACs axis, where it regulates Ca^2^⁺ transfer in cardiomyocytes [55]. DJ‐1 is another key component of the IP3Rs–GRP75–VDACs complex, modulating ER–mitochondrial Ca^2^⁺ flux by maintaining proper IP3R activity in M17 cells [34]. FUN14 domain containing 1 (FUNDC1), a classical MAMs protein that is conjugated to IP3R2, also accelerates Ca^2+^ translocation to mitochondria in cardiac muscle [56]. Additionally, cellular signals such as ROS exert an impact on the activity of IP3Rs [57]. ROS directly oxidizes cysteine residues within IP3Rs, thereby potentiating Ca^2+^ fluxes [58]. Other tethering proteins or complexes are also crucial for modulating Ca^2+^ transfer. For example, mitofusin2 (MFN2), a protein situated in OMM, is proposed as a tether for regulating Ca^2+^ transport at MAMs [59]. As a dynamin‐like protein, it functions to link the ER and mitochondria, analogous to its counterpart mitofusin1 (MFN1) [60]. Deletion of MFN2 results in the loss of MAMs, diminishing the efficiency of mitochondrial Ca^2+^ uptake [61]. PTPIP51 is also significant in mitochondrial Ca^2+^ uptake from the ER by interacting with vesicle‐associated membrane protein‐associated protein B (VAPB) [62]. Mutant VAPB linked to amyotrophic lateral sclerosis enhances Ca^2^⁺ transfer into mitochondria [62]. PDZ domain‐containing protein 8 (PDZD8), an ER‐resident protein, is as well involved in maintaining ER‐dependent mitochondrial Ca^2+^ homeostasis [63]. Concentrated in MAMs, PDZD8 is essential for MAMs formation in mammalian cells and requisite for mitochondrial Ca^2+^ uptake [63]. In summary, MAMs‐regulated Ca^2+^ transfer is of vital importance and closely related with physiological activities.

Recent investigations have revealed the correlation of cellular health with the proper regulation of Ca^2+^ signaling pathways mediated by MAMs. Thus, we will discuss the recent progress in understanding the mechanisms underlying Ca^2+^ signaling dysregulation and its contribution to pathogenesis, while also centering on diverse pathological endpoints where novel understandings of ER–mitochondrial Ca^2+^ transfer dysfunction have recently emerged [64]. Optimal mitochondrial Ca^2^⁺ levels support ATP production and cell viability, while also regulating various mitochondrial processes, including enzyme activities and substrate transporter functions [65, 66]. Specifically, it could enhance the efficiency of the tricarboxylic acid (TCA) cycle and the electron transport chain (ETC), thereby promoting ATP production [67]. In addition, Ca^2+^ transfer also modulates the shuttles of diverse nucleotides, metabolites, and cofactors across the IMM through the regulation of the Ca^2+^‐binding mitochondrial carriers (CaMCs) located within this membrane [66]. However, excessive mitochondrial Ca^2+^ uptake, representing a double‐edged sword, will lead to Ca^2+^ overload and cause damage to cellular health, rendering the organelles more susceptible to apoptotic stimuli [68]. Ca^2+^ signaling affects cell death via multiple pathways [68]. Sarco/endoplasmic reticulum calcium‐ATPases (SERCAs), the main ER Ca^2+^ uptake pump clustered in MAMs, has its function in orchestrating apoptosis modulated by various proteins. For instance, p53 in MAMs can alter the redox state of SERCAs, causing Ca^2+^ overload [69], while Bcl‐2 can downregulate SERCAs activity [70]. Excessive Ca^2+^ also acts as a trigger for mPTP opening [71]. Once the Ca^2+^ accumulation exceeds a certain threshold, it leads to mPTP opening, mitochondrial dysfunction, and ultimately cell death [72, 73, 74]. IP3Rs–GRP75–VDACs–MCU complex, the main Ca^2+^ transfer structure, is regulated by oncoproteins [75]. PTEN promotes Ca^2+^ transport to mitochondria by binding to IP3R, and its malfunction in cancers can lead to apoptosis resistance [75, 76]. Bcl‐2 and BAP1 also interact with IP3R and VDAC, respectively, affecting Ca^2+^ transfer and apoptosis regulation [77, 78, 79, 80]. In the condition of ER stress, ER and mitochondria change conformation and distribution, respectively, influencing their Ca^2+^ crosstalk and ATP production. The function of IP3R can be suppressed or enhanced under different ER stress conditions [81]. Other intracellular parameters, such as the physical distance of ER–mitochondria contact sites, can affect Ca^2^⁺ accumulation between these organelles [82]. In certain cancers, an increased ER–mitochondria distance helps to protect cells from mitochondrial Ca^2^⁺ overload and subsequent cell death [83, 84]. To sum up, the proper Ca^2^⁺ transport is essential for maintaining organ homeostasis and health, whereas uncontrolled Ca^2^⁺ overload serves as a potential trigger for diseases.

### 3.2 Synergetic Regulatory of Lipids

MAMs also play a critical role in controlling both the biosynthesis of phospholipids and their intermembrane transfer between the ER and mitochondria [85]. MAMs contain a variety of lipid transporters and biosynthetic enzymes, including phosphatidylethanolamine N‐methyltransferase 2 (PEMT2), fatty acid CoA ligase 4 (FACL4/ACSL4), cholesterol acyltransferase/sterol O‐acyltransferase 1 (ACAT1), phosphatidylserine synthase 1/2 (PSS1/2), and so on [86]. Throughout this intricate process, diverse intermediates are reciprocally transmitted multiple times between the two organelles. For instance, transfer of lipids like cardiolipin (CL), phosphatidylserine (PS), and phosphatidylethanolamine (PE) occurs at MAMs [21] (Figure 2B). PSS1 and PSS2, prototypical synthetic enzymes highly enriched in MAMs, play a central role in PS synthesis, with PSS1 transforming phosphatidylcholine (PC) and PSS2 converting PE into PS [87]. Additionally, PE import depends on the conversion of transported PS in MAMs to PE by PS decarboxylase (PSD) in mitochondria rather than direct import [88]. Indeed, the transfer of PS to mitochondria constitutes a rate‐limiting step in PE synthesis. This process has been empirically verified to be associated with a constellation of proteins, namely, oxysterol‐binding protein‐related proteins 5 and 8 (ORP5/8), MFN2, and CDP‐diacylglycerol synthase‐2 (CDS2), all of which are localized within MAMs. Furthermore, mitochondrial PE is derived from MAMs and subsequently methylated to PC by PE‐N‐methyltransferase (PEMT) [89]. In addition to the lipid synthetic mechanisms, enzymes responsible for cholesterol synthesis are also present on MAMs [90]. As an illustrative example, caveolin‐1 (CAV1) has been demonstrated to regulate cholesterol transport between the ER and mitochondria by binding to VDAC2 [91]. ACAT1 simultaneously converts free cholesterol into cholesterol esters, sustaining the dynamic equilibrium of cholesterol within the cells [92]. Notably, changes in cellular cholesterol levels can influence ER–mitochondria connectivity. Cholesterol depletion, in particular, has been shown to enhance the extent of these contacts [93]. Overall, impairments in MAMs‐mediated lipid biosynthesis and interorganelle transport can result in abnormal lipid metabolism and mitochondrial dysfunction, processes that are closely linked to diverse pathologies. The accumulated cholesteryl esters have a profound influence on the proliferation and metastasis of cancer cells [94]. PACS‐2, present in MAMs, has been demonstrated to regulate the formation of ER‐lipid synthesizing sites [95]. PACS‐2 knockdown markedly reduces the levels of ACSL4 and PSS1 in the MAMs, whereas its overexpression increases their abundance [96]. Additionally, overexpression of GRP75 or MFN2 strengthens MAMs integrity and promotes cholesterol ester synthesis, ultimately leading to increased intracellular cholesterol storage [97]. In AD patients, MAMs are significantly enhanced, and its number is increased, facilitating phospholipid production and interorganelle transfer [98].

Among the phospholipids, CL is uniquely synthesized in the IMM, and PE is predominantly synthesized within mitochondria [99]. CL is synthesized through a series of modifications of phosphatidic acid (PA), which is produced by several distinct enzymes residing in the ER, OMM, and potentially IMM by acylglycerol kinase [100, 101]. Recent studies have demonstrated that the VAPB‐PTPIP51 complex regulates PA transfer in MAMs [102]. As a characteristic mitochondrial protein, CL governs diverse mitochondrial functions, including the organization of the respiratory chain, modulation of mitochondrial dynamics, and apoptosis [103]. Primarily located in the IMM, CL stabilizes mitochondrial respiratory chain complexes, supports efficient oxidative phosphorylation (OXPHOS) activity, and maintains membrane integrity and cristae morphology [104, 105]. Under cellular stress, CL is externalized to the OMM, where it serves as a signaling molecule to promote mitophagy and apoptosis [106, 107]. Further study has revealed that, upon mitophagy stimulation, CL is enriched at MAMs, where it associates with MFN2 and other proteins involved in autophagosome formation [108]. Additionally, CL also regulates mitochondrial dynamics by interacting with DRP1, promoting its oligomerization, which is critical for mitochondrial fission [109, 110]. Simultaneously, mitochondrial fusion is associated with CL, which is necessary for Optic atrophy 1 (OPA1) dimerization and IMM fusion [111]. And the accumulation of oxidized CL in the OMM can recruit and activate caspase‐8, triggering mPTP opening and the release of cytochrome *c*, thereby initiating programmed cell death [112] (Figure 2C). PE plays a vital role in regulating mitochondrial dynamics and the biogenesis of OMM proteins [113, 114]. In conclusion, the associations of CL and PE with other mitochondrial events highlight the complex interplay among phospholipid dynamics, mitochondrial morphology, and significant cellular processes under healthy conditions.

### 3.3 Stress Signal Transduction Regulation

**FIGURE 3:**
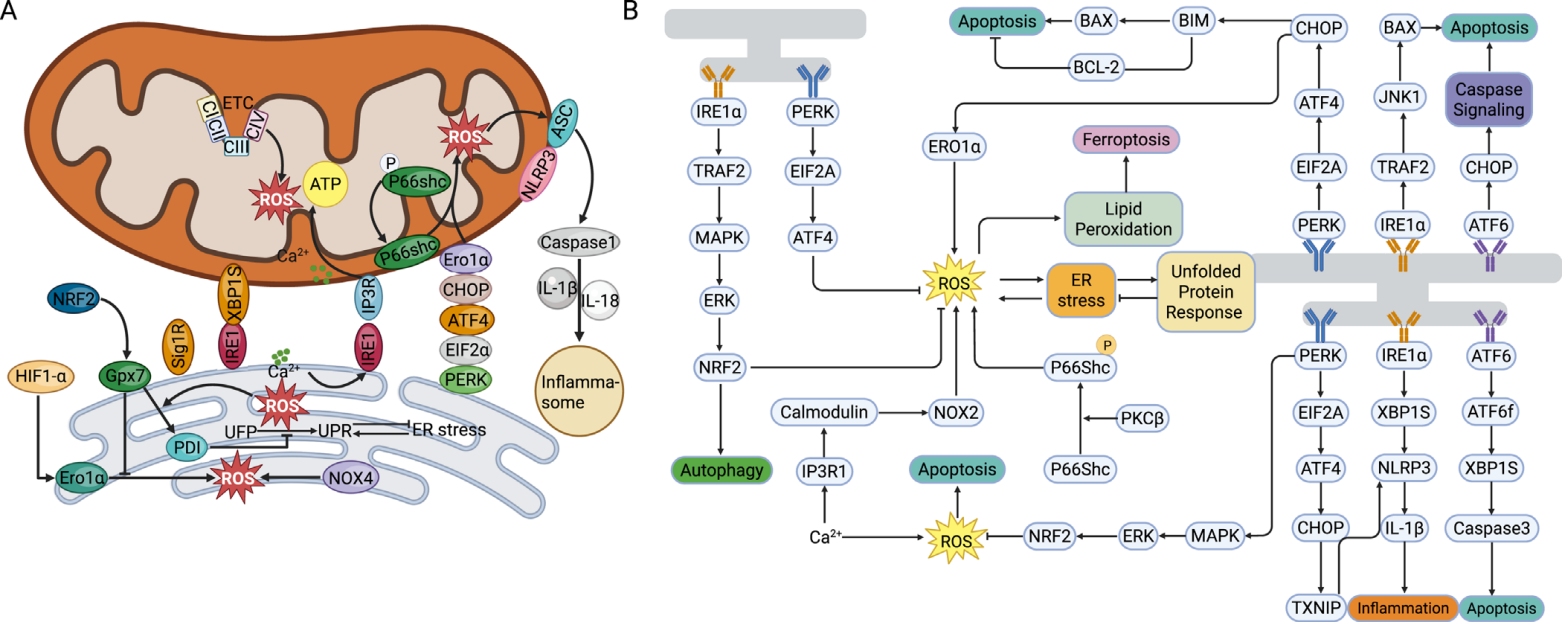
Molecular modulation of ER stress response. (A) Components regulating ER stress response and oxidative stress. Under conditions of ER stress, the UPR is activated to restore protein homeostasis and support cell survival. The accumulation of unfolded or misfolded proteins in the ER lumen prompts GRP78 to dissociate from the ER stress sensors—PERK, ATF6, and IRE1α—as it binds to the misfolded proteins. This dissociation triggers the activation of each sensor, initiating their respective downstream signaling pathways. ROS, closely associated with ER stress and oxidative stress, is also regulated by MAMs. Additional ER stress stimulates the production of ROS, thus amplifying the oxidative stress. Ero1α and P66Shc are enzymes associated with ROS sources located in MAMs. In hypoxia conditions, Ero1α also modulates the communication of Ca^2+^ in MAMs. In addition, NOX4 has also been proved to have a crucial role in the formation of ROS. Cellular ROS disrupts binding of thioredoxin to the thioredoxin‐interacting protein (TXNIP), leading to the accumulation of free TXNIP, which binds to and transports NLRP3 to the MAMs, promoting assembly of an active inflammasome complex (created in https://BioRender.com). (B) Molecular network of ER stress response. Excessive ROS production prompts ER stress and triggers UPR to restore ER homeostasis. ER stress also activates NADPH oxidase, which contributes to the uncoupling of nitric oxide synthase (NOS) and promotes the generation of reactive oxygen species (ROS). In addition, as summarized before, Ca^2+^ is also associated with ROS production. ER stress induces the UPR which has three branches, mediated by PERK, IRE1α and ATF6 to combat dyshomeostasis. Dimerization and phosphorylation of PERK and IRE1α initiate their respective signaling cascades: the PERK–eIF2α–ATF4 axis and the IRE1α‐mediated splicing of XBP1 mRNA to generate XBP1s, to promote cell survival. Ero1α and P66shc, which are in MAMs, regulate the production of ROS and engage in ER stress as contributory factors (created in https://BioRender.com).

As a central quality‐control hub, the ER maintains protein homeostasis and cellular health by facilitating proper protein folding through the action of resident enzymes. ER stress arises from the buildup of misfolded or unfolded proteins. While the unfolded protein response (UPR) is initially activated to restore proteostasis, prolonged UPR signaling can become maladaptive [115, 116]. MAMs potentially serve as a vital contact site, regulating UPR and ER stress to modulate cell fates. The UPR is principally initiated and regulated by three enzymes: inositol‐requiring enzyme 1 (IRE1), double‐stranded RNA‐activated protein kinase R‐like ER kinase (PERK), and activating transcription factor 6 (ATF6). Under resting conditions, IRE1, PERK, and ATF6 are maintained in an inactive form through the interaction of with the ER chaperone glucose‐regulated protein 78 (GRP78). Accumulation of unfolded proteins competes with the GRP78‐binding receptor, activating these three sensors and initiating the UPR [117] (Figure 3A). Under stress, three main UPR pathways are activated respectively by IRE1 and PERK, which both locate in MAMs, as well as ATF6. The unfolded protein binds to PERK, triggering PERK to multimerize and phosphorylate itself [118]. Subsequently, PERK activation phosphorylates and inactivates eukaryotic translation initiation factor 2α (EIF2A), thereby reducing protein synthesis [119]. The translation of ATF4 mRNA regulated by phosphorylated EIF2A activates the C/EBP‐homologous protein (CHOP) and other genes involved in autophagy, antioxidant responses, and cell death [120, 121]. Recently, Bassot et al. have examined the PERK interactome and identified ERO1α as its binding enzyme, helping to maintain bioenergetics and suppress oxidative stress, ultimately reducing ER stress [122]. PERK‐deficient mouse embryonic fibroblasts (MEFs) show reduced MAMs formation, leading to diminished ER–mitochondrial Ca^2^⁺ and ROS signaling, ultimately, attenuated apoptosis [123]. Another UPR‐related mechanism involves p38 mitogen‐activated protein kinase (p38 MAPK) activation, which activates CHOP and promotes ER stress‐mediated apoptosis by enhancing ROS production [124]. Upon ER stress, IRE1 is released from GRP78 and autophosphorylates, which triggers its endoribonuclease activity to process X‐box‐binding protein 1 (XBP1) mRNA [125, 126]. ATF6 translocates to the Golgi apparatus and is cleaved by site‐1 and site‐2 proteases [127]. After cleavage, ATF6 releases a cytosolic fragment (ATF6f) that directly controls the transcription of XBP1 [128] (Figure 3B). Sigma‐1 receptor (Sig‐1R), another protein residing in MAMs, is highly expressed in the central nervous system. In rest state, Sig‐1R remains inactive in complex with GRP78. ER stress triggers its release and translocation, reducing UPR activation. Overexpression of Sig‐1R counters ER stress by inhibiting PERK and ATF6, whereas knockdown leads to MAMs disruption, mitochondrial impairment, and enhanced apoptotic signaling [129].

**FIGURE 4:**
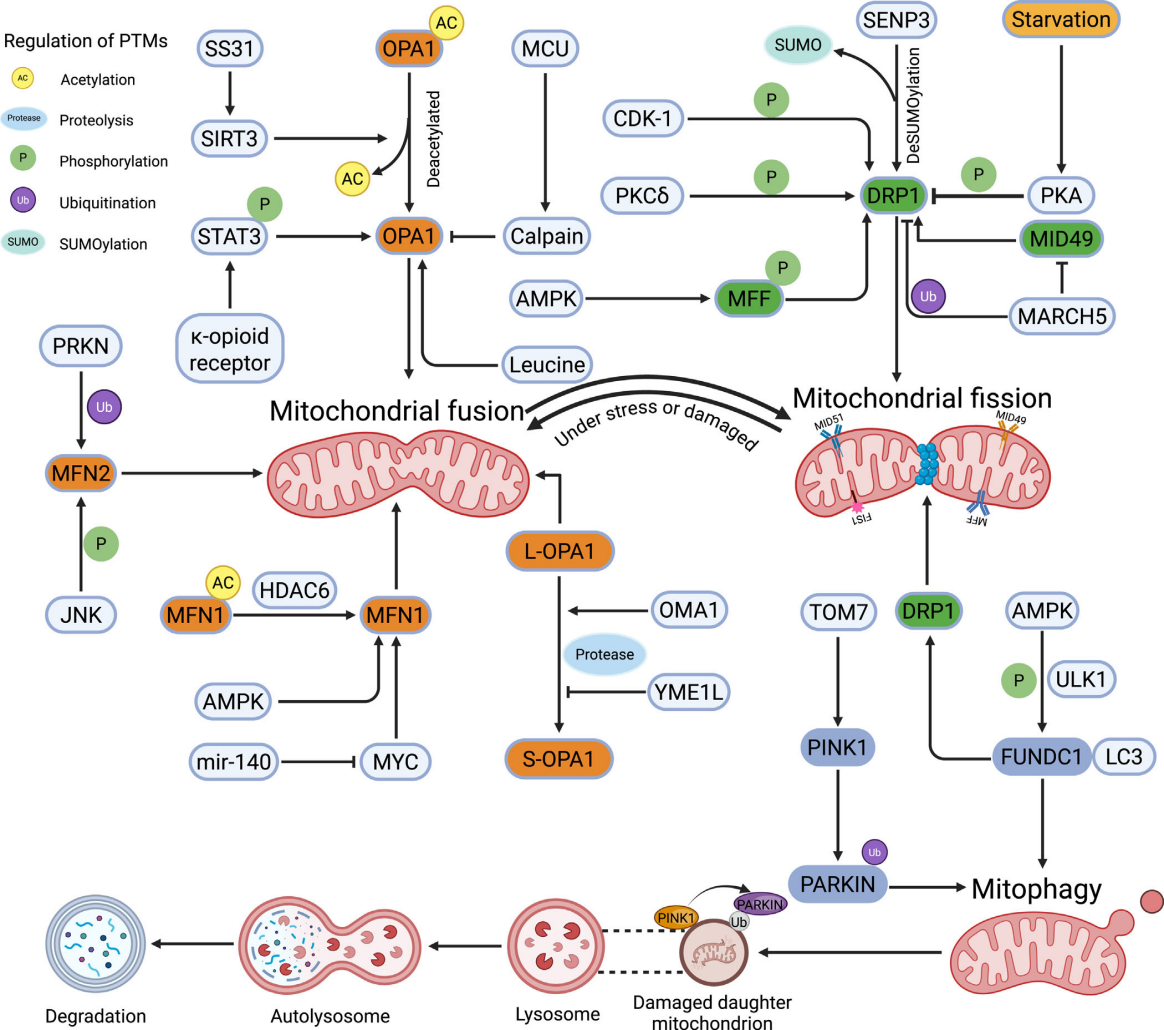
Molecular modulation of mitochondrial dynamics and mitophagy. Mitochondria are highly dynamic organelles that maintain genomic integrity and metabolic homeostasis by continuously remodeling their network through a tightly regulated balance between fission and fusion, processes orchestrated by evolutionarily conserved molecular machineries. The processes of mitochondrial fusion, fission, and mitophagy are closely interconnected. Briefly speaking, mitochondrial fusion facilitates the mixing and exchange of intramitochondrial contents between individual mitochondria, thereby contributing to the maintenance of mitochondrial function and overall cellular homeostasis; while mitochondrial fission plays a pivotal role in mitochondrial quality control by segregating damaged or dysfunctional mitochondria, thereby facilitating their removal via mitophagy. Additionally, it promotes apoptotic signaling in response to severe cellular stress. Key enzymes like OPA1, MFN1 and MFN2, DRP1, PINK1, and FUNDC1, which involved in these processes, are mainly regulated by diverse PTMs, such as phosphorylation, SUMOylation, ubiquitination, acetylation and S‐nitrosylation modification, which displayed in the top left corner (created in https://BioRender.com).

MAMs also play a key role in regulating oxidative stress, which arises from an imbalance in cellular redox status due to ROS accumulation, serving as a hub linking ER stress and oxidative stress. To counteract the oxidative stress, the UPR pathways are initiated as an adaptive modality for the preservation of cell functionality. Nevertheless, supplementary ER stress augments the generation of ROS, consequently magnifying the oxidative stress [130]. In accordance with prior research, enzymes situated in MAMs, namely, Ero1α and the 66 kDa subtype of growth factor adapter Shc (P66shc), constitute alternative sources of ROS in addition to ER stress (Figure 3B). Ero1α fabricates disulfide bonds, which are essential for protein folding and stress alleviation, and concomitantly transfers electrons to molecular oxygen, with ROS being produced as a concomitant byproduct [131]. The elevated expression of Ero1α is intimately associated with the overproduction of ROS, precipitating cell apoptosis [39]. Additionally, P66shc, a cytoplasmic adaptor protein, is also correlated with oxidative stress. Under oxidative stress conditions, P66Shc undergoes phosphorylation, isomerization, and dephosphorylation before relocating to MAMs to generate ROS [132]. To sum up, ER stress and oxidative stress regulated by MAMs are vital for maintaining cellular equilibrium in cells. Dysfunction in this process can disrupt the normal communication between organelles and contribute to various pathological conditions.

### 3.4 Regulation of Mitochondrial Dynamics and Mitophagy

As dynamic organelles, mitochondria preserve the integrity of their genome and metabolic function by modulating their network architecture via a strictly regulated balance between fission and fusion. These are intricate processes that are elaborately governed by conserved molecular machineries [133]. Mitochondrial fusion serves to blend the intramitochondrial constituents among mitochondria, thereby facilitating the maintenance of mitochondrial functions. In contrast, mitochondrial fission plays a crucial role in mitochondrial quality control by eradicating damaged or malfunctioning mitochondria and stimulates mitophagy to cope with severe cellular stress [134] (Figure 4). Mitochondrial fusion requires coordination of OMM fusion and IMM fusion [135]. This two‐step process necessitates the involvement of three GTPases, namely, MFN1, MFN2, and OPA1 [136]. To be specific, OMM fusion is carried out by MFN1 and MFN2, whereas IMM fusion depends on OPA1 [137]. The most efficient mitochondrial fusion process occurs when both MFNs are concurrently present. The activity of MFNs is finely regulated by post‐translational modifications (PTMs). For instance, acetylation at K222 or K491 inhibits the MFN1 GTPase activity, whereas histone deacetylase 6 (HDAC6)‐mediated deacetylation under glucose deprivation enhances MFN1 function and drives mitochondrial hyperfusion [138]. PRKN‐dependent ubiquitination of MFN2 regulates MAMs remodeling and promotes mitophagy, while stress‐induced Jun N‐terminal kinase (JNK) phosphorylation of MFN2 leads to HUWE1‐mediated proteasomal degradation [139, 140]. OPA1, the principal regulator of the IMM, is also subject to several PTMs. Studies have also shown that OPA1, as an acetylation‐modified protein, is targeted by SIRT3 deacetylation to modify its activity [141, 142]. While mitochondrial fission is a multistep process that results in the division of a single mitochondrion into two separate organelles [137]. Mitochondrial fission primarily occurs at OMM constriction sites coordinated with the ER. This process involves the recruitment of DRP1 by several OMM adaptor proteins, including mitochondrial fission 1 (FIS1), mitochondrial fission factor (MFF), and mitochondrial dynamics proteins MID49 and MID51 [136, 143]. These proteins are all known to be localized at MAMs. Several other MAMs‐related proteins are also engaged in the process of mitochondrial fission. A variety of PTMs that occur in MAMs are also implicated in the modulation of DRP1 activity [144]. Phosphorylation of DRP1 at serine 616 by CDK1 and PKCδ enhances its activity, thereby facilitating mitochondrial division during cellular processes [145, 146]. Conversely, when DRP1 undergoes phosphorylation at S637 mediated by protein kinase A (PKA), its activity is decreased. And dephosphorylation of DRP1 at S637 via calcineurin‐dependent signaling promotes mitochondrial fission by facilitating DRP1 translocation to the OMM [147]. SUMOylation also regulates the activity of DRP1. SUMO‐1‐mediated SUMOylation of DRP1 prevents its lysosomal degradation, thereby enhancing its stability and further promoting mitochondrial fission. On the other hand, deSUMOylation of DRP1 by SUMO‐specific protease 3 (SENP3) enhances the mitochondrial fission by promoting the interaction between DRP1 and MFF [148]. Phosphorylation of MFF by AMPK enhances DRP1 translocation to the OMM [149, 150]. Furthermore, the E3 ubiquitin ligase MARCH5 ubiquitinates DRP1 and its receptor MID49, targeting them for proteasomal degradation and thereby restricting mitochondrial fission [151] (Figure 4).

Mitophagy is intimately linked to mitochondrial fission, as a dysfunctional mitochondrion requires segregation from the mitochondrial network via fission prior to clearance by mitophagy which represents a complicated and dynamic process [152]. Depending on the tag associated with the damaged portion targeted for elimination, mitophagy can be classified into either the PINK1/Parkin‐mediated pathway or the receptor‐dependent pathway, which are both modulated by MAMs [153]. Parkin‐mediated ubiquitination accelerates the degradation of various OMM proteins, including MFN1/2 and VDAC1, while also recruiting autophagy receptors such as p62 and optineurin. These ubiquitinated proteins are then phosphorylated by PINK1, promoting further Parkin recruitment to mitochondria and amplifying ubiquitin chain formation [154]. As a consequence, the ubiquitinated mitochondria are recognized by autophagy receptors, ultimately leading to the formation of autophagosomes that eliminate the damaged mitochondria [155]. With respect to receptor‐dependent mitophagy, mitophagy receptors endowed with a LC3‐interacting region (LIR) facilitate the direct targeting of damaged mitochondria by LC3‐dependent autophagosomes [156]. FUNDC1, a prototypical mitophagy receptor that resides in MAMs, is capable of directly instigating mitophagy by binding to LC3 [157]. Under pathological conditions such as hypoxia or ER stress, mitophagy helps to maintain cellular balance by clearing damaged mitochondria from the healthy network and preventing mitochondrial apoptosis. FUNDC1 interacts with LC3B and functions as a mitophagy receptor to initiate the process [158, 159]. Mutations or deletions of the LIR motif disrupt FUNDC1‐mediated mitophagy [160]. The phosphorylation of FUNDC1 critically determines its binding affinity for LC3, thereby regulating mitophagy induction. AMPK recruits the autophagy initiator ULK1 to phosphorylate FUNDC1 at Ser17, strengthening its interaction with LC3B [161]. Conversely, PGAM family member 5 (PGAM5) promotes mitophagy by dephosphorylating FUNDC1 at Ser13 [162]. In conclusion, MAMs orchestrate mitochondrial fission and fusion and serve as hubs for molecules that initiate mitophagy. Their proper function is essential for maintaining mitochondrial integrity and consequently, has a profound impact on overall health and diseases.

## 4 Roles of MAMs in Diseases

Owing to the diverse functions of MAMs, it is widely acknowledged that MAMs are intimately correlated with physiological processes. Moreover, the dysregulation of MAMs can act as either a causative factor or a resultant outcome of a broad spectrum of common diseases, such as cancers, neurodegenerative diseases, metabolic diseases and cardiovascular diseases.

### 4.1 MAMs and Cancers

**FIGURE 5:**
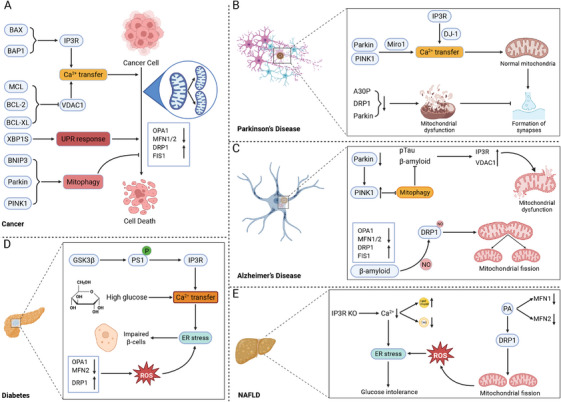
Interplay between MAMs and common diseases. Accumulating evidence has indicated that MAMs are closely associated with the progression of diverse common diseases such as cancers, neurodegenerative diseases, metabolic diseases, and cardiovascular diseases. Intercellular processes including Ca^2+^ transfer, lipid metabolism, ER stress response, mitochondrial dynamics, and mitophagy are all regulated by the components of MAMs and play crucial roles in the development and progression of diseases (created in https://BioRender.com). (A) Cancers. The Ca^2+^ transfer regulated by IP3R–GRP75–VDAC1 complex serves as the main regulator of the development of cancers. And oncogenic conditions are potent triggers of ER stress, with UPR activation facilitating oncogenic transformation through all signaling branches, thereby promoting tumor growth and immune evasion. In addition, the expression of DRP1 and FIS1 is upregulated in the development of cancers. (B) Parkinson's disease. PD, featured with degeneration of substantia nigra neurons, is closely linked with MAMs. PINK1 and Parkin, contributing to ER–mitochondria tethering, are associated with PD. Under conditions lacking Parkin and PINK1, MAMs are perturbed by abnormal Ca^2+^ level, leading to the development of PD. What is more, DRP1 deficiency also impairs mitochondrial distribution to synapses and disrupts synaptic function. (C) Alzheimer's disease. In fibroblasts of AD patients, the number of MAMs and lipid transfer are significantly upregulated. In addition, expression levels of OPA1 and MFN1/2 are significantly lower, whereas DRP1 and FIS1 levels are higher in AD patients. (D) Diabetes. Several proteins located at MAMs are crucial for insulin signaling, thus regulating the progression of diabetes. GSK3β, a crucial inhibitor of glycogen synthase, interacts with and potentiates IP3R activity, sustaining proper Ca^2+^ transfer. Pancreatic β‐cell dysfunction can also be initiated by ER stress. (E) Nonalcoholic fatty liver disease (NAFLD). NAFLD is a metabolic syndrome featured with aberrant lipid accumulation in the liver. ER stress response, which is linked with Ca^2+^ homeostasis, leads to a decrease in protein folding and causes damage to glucose tolerance. In addition, reduced mitochondrial fragmentation may lead to excessive fat accumulation.

Recent research increasingly supports the critical involvement of MAMs in the regulation of tumorigenesis and cancer progression [163]. In the processes of cancer development, Ca^2+^ transfer exerts a dual role. In cancer cells, ER–mitochondrial Ca^2+^ transfer is often suppressed to prevent apoptotic cell death, while elevated Ca^2^⁺ flux can facilitate metabolic reprogramming and enhance migratory capacity [164]. As the primary regulators of the Ca^2^⁺ transfer axis, IP3Rs exert divergent roles across cancer models. In MCF‐7 breast cancer cells and non–small‐cell lung cancer cells, IP3R suppression leads to autophagic cell death [165, 166] (Figure 5A). On the contrary, DT40 and HeLa cell lines deficient in all three IP3R isoforms have been successfully generated and are capable of surviving [167, 168]. Oncogenic proteins can also promote IP3R function to sustain cell survival. For example, the antiapoptotic protein Bcl‐XL can sensitize IP3Rs to IP3, thereby promoting ER–mitochondrial Ca^2^⁺ transfer and facilitating prosurvival signaling [169]. In addition, other proteins such as protein kinase B (PKB/AKT), NADPH oxidase 4 (Nox4), phosphatase and tensin homolog (PTEN), BRCA1‐associated protein 1 (BAP1), STAT3, and B‐cell lymphoma 2 (Bcl‐2) all directly and indirectly target IP3R and regulates its activity, thus contributing to the process of Ca^2+^ transfer [170, 171, 172]. Similarly, other components of Ca^2+^ transfer axis like VDAC1, is also dysregulated in cancers. Antiapoptotic proteins such as Bcl‐2, Bcl‐XL, and Mcl‐1 can associate with VDAC1 at MAMs, inhibiting mitochondrial Ca^2^⁺ uptake and thus preventing mitochondrial Ca^2^⁺ overload, functioning as key modulators of cell survival [80]. In addition to the regulation of IP3Rs–GRP75–VDACs complex, mitochondrial Ca^2^⁺ uptake is as well controlled by local SERCA pump, which is negatively regulated by the MAMs‐resident oxidoreductase TMX1. In xenografts of mice, reduced TMX1 levels enhance SERCA activity and diminish mitochondrial Ca^2^⁺ transfer, as a result promoting tumor growth [173]. In addition, oncogenic conditions frequently trigger ER stress, and UPR activation supports malignant transformation by promoting tumor growth, vascularization, and immune escape [174]. Targeting the UPR, through either its inhibition or the enhancement of ER stress, could effectively limit oncogenesis. Notably, high XBP1s levels in biopsies from brain, breast, and hematologic malignancies correlate with unfavorable outcomes and shorter survival [175, 176]. The IRE1α‐XBP1 UPR branch collaborates with hypoxia‐inducible factor 1α (HIF1α) in human triple‐negative breast cancers to promote angiogenesis and tumor cell proliferation [176]. Additionally, activation of the IRE1α‐XBP1 pathway promotes prostate cancer progression via MYC signaling and contributes to hepatocellular carcinoma by enhancing metabolic inflammation [177, 178]. And inhibition of EIF2α‐mediated translational suppression can selectively induce cytotoxicity in aggressive prostate cancer cells [179]. Oncogenic signaling frequently disrupts the equilibrium between mitochondrial fusion and fission [180]. For example, DRP1‐mediated mitochondrial fission is essential for KRAS‐mediated transformation in pancreatic cells, whereas in hepatocellular carcinoma, FIS1 phosphorylation induces mitochondrial fragmentation, thereby facilitating metastasis [181, 182]. Conversely, the fusion protein MFN2 has been implicated in regulating angiogenesis and metastasis in cancers; notably, MFN2 overexpression in endothelial cells suppresses angiogenesis by downregulating proangiogenic factors [182, 183]. Accumulation of dysfunctional mitochondria caused by dysregulated mitophagy is also involved in tumorigenesis [184]. The absence of Bnip3 in mice leads to faster tumor progression relative to wild‐type animals, a phenomenon associated with excessive buildup of damaged mitochondria and elevated ROS [185]. Expression of Parkin and PINK1 are also found lost in in many types of cancer [186].

### 4.2 MAMs and Neurodegenerative Diseases

Neurodegenerative diseases comprise a spectrum of disorders defined by the chronic and progressive loss of neural tissue. Recent evidence indicates that MAMs dysfunction plays a critical role in their pathogenesis [187, 188]. Herein, we will focus on the involvement of MAMs in three common types including Parkinson's disease (PD), Alzheimer's disease (AD), and Huntington disease (HD) and we will detailedly talk about how proteins situated at MAMs are regulated and operate in the context of neurodegenerative pathology.

#### 4.2.1 Parkinson's Disease

PD is characterized by the degeneration of neurons in the substantia nigra, leading to tremors, gait abnormalities, and muscle rigidity [189]. α‐Synuclein, the primary component of Lewy bodies in PD, localizes to MAMs, while the pathogenic mutant form‐A30P exhibits reduced MAMs association [190]. Moreover, PINK1 and Parkin, both contributing to ER–mitochondria tethering, are linked to recessive familial forms of PD [191]. Loss of Parkin, whether through genetic knockout in mice or pathogenic mutations in humans, perturbs MAMs structure and results in dysregulated Ca^2^⁺ signaling [191]. Mitochondrial rho GTPase 1 (Miro1), an OMM protein, is implicated in PD through its interactions with PINK1 and Parkin [192] (Figure 5B). Miro proteins serve as Ca^2+^‐dependent docking sites for Parkin recruitment [192]. Another PD‐related protein DJ‐1, strongly associated with MAMs, is involved in multiple cellular processes, especially Ca^2^⁺ homeostasis [193]. DJ‐1 ablation leads to aggregation of IP3R3 at MAMs, causing its disassociation with the IP3R–GRP75–VDAC1 complex [34]. Parkin is also essential for ubiquitin‐dependent proteolysis and regulates mitochondrial health by modulating mitochondrial dynamics [194]. Mitochondrial dynamics are essential for the formation of synapses and dendritic spines in neurons. Inhibition of mitochondrial fragmentation reduces mitochondrial presence in dendritic spines and impairs synaptic development, whereas enhanced fission promotes synapse formation [195]. DRP1 deficiency impairs mitochondrial distribution to synapses and disrupts synaptic function [195]. Additionally, in drosophila model, Parkin mutations are associated with increased oxidative stress‐induced mitochondrial dysfunction, characterized by mitochondrial swelling and fragmented cristae [196]. These findings indicate that MAMs‐associated proteins influence PD progression through modulation of cellular functions, presenting potential targets for future pharmacological interventions.

#### 4.2.2 Alzheimer's Disease

AD features neuronal and synaptic loss, with hallmark abnormal β‐amyloid (Aβ) deposition and accumulation of hyperphosphorylated Tau protein (pTau) [197]. Exposure of primary hippocampal neurons to Aβ upregulates MAMs‐associated proteins like IP3R3 and VDAC1, increases the number of MAMs and enhances ER–mitochondrial Ca^2^⁺ fluxes in SH‐SY5Y cells [198]. Furthermore, MAMs and lipid transfer are significantly elevated in fibroblasts derived from AD patients [199] (Figure 5C). Normally, neurons of AD patient display unfavorable mitochondrial dysfunction due to the dysregulation of MAMs‐related proteins. In AD, OPA1 and MFN1/2 expression is markedly decreased, while DRP1 and FIS1 levels are elevated [200]. Nitric oxide generated in response to Aβ triggers DRP1 S‐nitrosylation, promoting mitochondrial fragmentation and neuronal damage, which may accelerate AD progression [201]. Mitochondrial dysfunction arising from aberrant mitophagy is also a key factor in AD pathogenesis. Ye et al. demonstrated that increased Parkin‐mediated mitophagy in mutant hAPP neurons and AD patients’ brain tissue [202]. Furthermore, as AD advances, cytosolic Parkin levels in patient brains decline, causing an abnormal buildup of PINK1 [203]. Restoring mitophagy promotes Aβ plaque reduction, tau phosphorylation attenuation, and cognitive function preservation [204, 205].

#### 4.2.3 Huntington Disease

HD is an autosomal dominant neurodegenerative disorder marked by motor dysfunction, psychiatric disturbances, and cognitive impairment [206]. The pathogenesis of HD is attributed to mutations in the gene encoding Huntington (HTT) [207]. Similar to other neurodegenerative disorders, Ca^2+^ signaling is dysregulated in HD [208]. Neuronal degeneration in HD is largely attributed to disrupted mitochondrial dynamics—particularly excessive fission—which impairs mitochondrial function, axonal transport, and synaptic transmission [209]. In HD patients, upregulation of FIS1 and DRP1, along with downregulation of MFN1/2 and OPA1, has been observed. These disruptions compromise mitochondrial function, hinder neuronal transport, and trigger cell death, potentially accelerating disease progression [210]. Excessive mitochondrial fission in HD striatum disrupts MAMs integrity, leading to impaired Ca^2^⁺ signaling and ROS imbalance [211]. Mitophagy defects also result in the accumulation of damaged mitochondria, promoting HD progression. Activation of the PINK1‐Parkin pathway preserves mitochondrial integrity and provides neuroprotection [212].

### 4.3 MAMs and Metabolic Diseases

Recent studies have implicated dysregulated MAMs‐associated proteins in the pathogenesis of metabolic diseases, revealing novel molecular mechanisms at the MAMs interface. In this section, we will discuss the role of MAMs in metabolic diseases including diabetes and nonalcoholic fatty liver disease (NAFLD).

#### 4.3.1 Diabetes

Diabetes is a metabolic disease marked by insulin resistance, defective insulin secretion, and abnormal glucose metabolism [213]. Multiple proteins essential for insulin signaling and cellular survival are localized at MAMs (Figure 5D). GSK3β, known for its role in glycogen synthase inhibition, interacts with and enhances the activity of IP3Rs [55]. In murine pancreatic β cells, GSK3β facilitates a basal ER–mitochondrial Ca^2^⁺ leak via PS1 phosphorylation [214]. Likewise, PDK4 at MAMs interacts with the IP3Rs–GRP75–VDACs complex to enhance ER–mitochondrial Ca^2^⁺ transfer [215]. Moreover, in rat‐derived pancreatic cells, acute glucose exposure stimulates the formation of MAMs and Ca^2^⁺ transfer, leading to ER Ca^2^⁺ store depletion and subsequent ER stress activation [216]. In pancreatic tissue from T2D patients, IP3R2‐VDAC1 interactions were reduced compared to healthy controls [217]. T2D β cells exhibit decreased VDAC1 and increased IP3R2 levels, reflecting distinctive modifications in MAMs‐related proteins associated with the disease [217]. The progression of diabetes is also closely associated with pancreatic β‐cell dysfunction, which can be initiated by ER stress [218]. DRP1 expression markedly enhances apoptosis triggered by ER stress in DRP1 wild‐type‐activated β cells [219]. Other proteins regulating mitochondrial dynamics are also associated with diabetes. Studies revealed that levels of MFN2 and OPA1 are reduced in skeletal muscles of Zucker fatty rats and T2D patients [220]. Inhibition of DRP1‐mediated mitochondrial fission reduces ROS production, decreases UPR‐related proteins, and alleviates mitochondrial depolarization, while also enhancing insulin sensitivity in placental cells under hyperglycemic conditions [218, 220].

#### 4.3.2 Nonalcoholic Fatty Liver Disease

NAFLD is a metabolic syndrome of unclear origin, primarily driven by excessive hepatic lipid accumulation [221]. Recently, growing evidence has indicated that MAMs‐regulated structural and bioenergetic mitochondrial alterations contribute to the pathogenesis of NAFLD (Figure 5E). Ca^2+^ channels serve as an important regulator in the progression of NAFLD and may represent a promising target for both diagnostic and therapeutic strategies in the future. In mice feeded with high‐fat diet (HFD), IP3R1 expression is increased in the MAMs, leading to mitochondrial dysfunction and the disruption of metabolic homeostasis [222]. On the contrary, IP3R1‐knockout mice exhibited a significant decrease in Ca^2+^ signaling and reduced triglycerides accumulation in the serum and liver [223]. A decline in Ca^2^⁺ concentration activates the ER stress response, compromising protein folding, chaperone function, and glucose tolerance. This metabolic disturbance subsequently stimulates the production of diacylglycerol and triacylglycerol [224]. NAFLD progression is closely associated with disrupted mitochondrial dynamics. In hepatocytes, PA treatment induces mitochondrial fragmentation, promotes cytochrome *c* release, and elevates ROS production [225]. In HFD‐induced NAFLD models, liver tissue exhibits increased DRP1 expression, mitochondrial fragmentation, and enhanced hepatocyte lipolysis. Inhibiting mitochondrial fission mitigates oxidative stress and hepatic dysfunction caused by excess fat intake, thereby protecting against steatosis [226, 227]. Additionally, mitophagy promotes lipid clearance, and its activation alleviates hepatic steatosis, thereby slowing NAFLD progression [228, 229]. In the early stage of the long‐term NAFLD mouse model, PINK1/Parkin‐dependent mitophagy is activated to mitigate hepatic lipid overload [230].

### 4.4 MAMs and Cardiovascular Diseases

In the mammalian heart, cardiomyocytes comprise the majority of tissue volume, while endothelial cells, fibroblasts, and vascular smooth muscle cells (VSMCs) constitute the remainder [231]. The heart's function relies on the integrity of these specialized cells and their sarcomeric structures [232]. Any dysregulation in specific proteins located at MAMs has the potential to modify the cardiac functions.

#### 4.4.1 Myocardial Ischemia/Reperfusion

**FIGURE 6:**
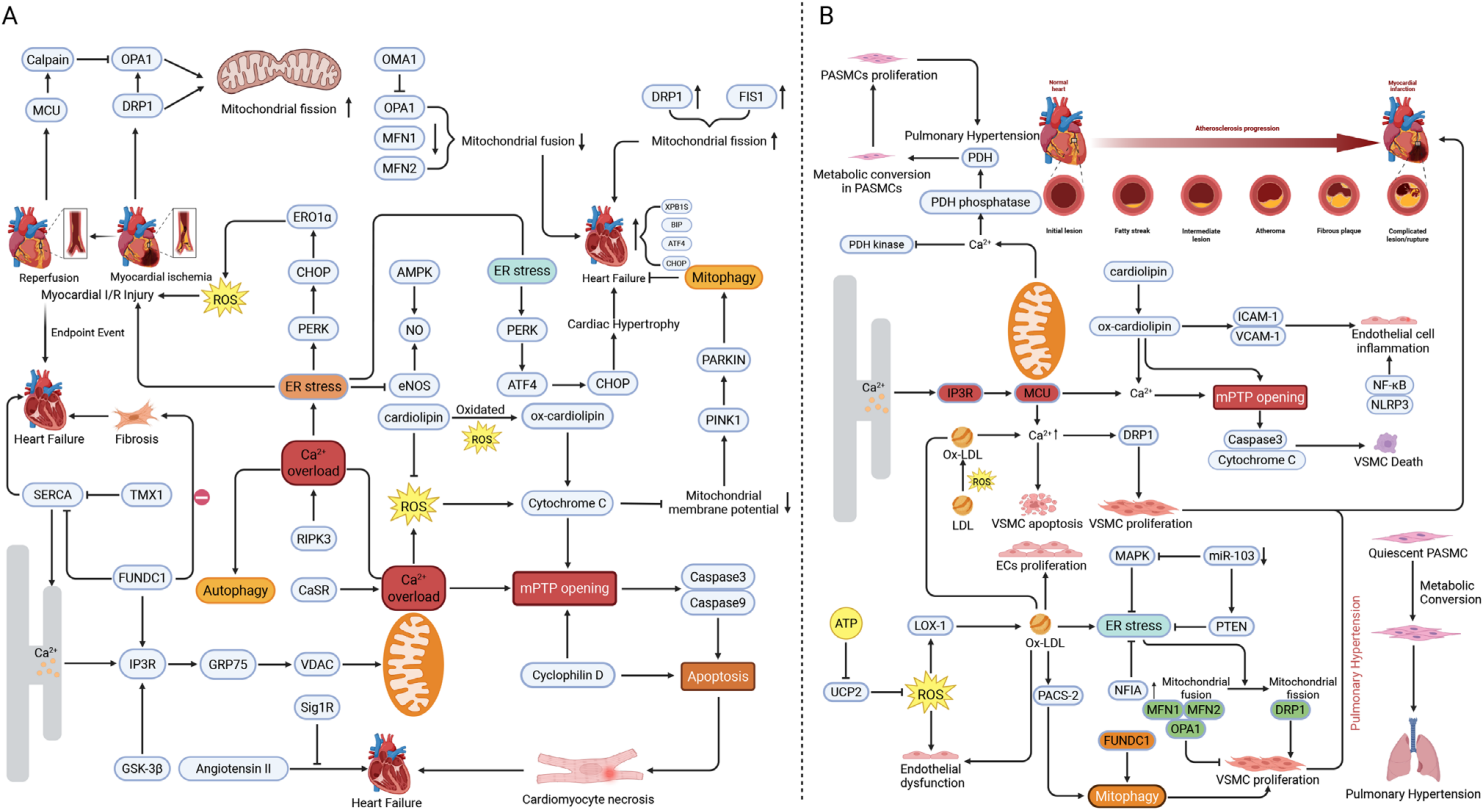
Interplay between MAMs and cardiovascular diseases. Cardiovascular pathologies, including I/R injury, heart failure, atherosclerosis and pulmonary hypertension, lead to the dysregulation of MAMs. And disordered MAMs are also closely associated with progression of cardiovascular diseases due to their regulation of diverse cellular processes (created in https://BioRender.com). (A) Heart diseases. Cardiomyocyte death during reperfusion damage is induced by the mPTP opening caused by Ca^2+^ overload. Oxidative stress induced by excessive ROS production also contributes to the development of myocardial I/R injury. Accordingly, UPR is activated during I/R and serves as protective factor to deal with ER stress. In addition, ischemia is accompanied by mitochondrial fragmentation, which is then degraded through mitophagy to sustain myocardial fitness. Heart failure is the endpoint of most heart diseases and is similarly linked with the cellular events regulated by MAMs. (B) Vascular diseases. Featured with uncontrolled VSMCs phenotypic transformation, the progression of atherosclerosis is induced by excessive Ca^2+^ and accumulated oxidized CL. And ROS can stimulate VSMCs proliferation and induce apoptosis in PASMCs. In addition, ROS relates to mitochondrial dynamics, leading to a shift in mitochondrial state from fusion to fission. The imbalance of mitochondrial dynamics leads to unregulated VSMC phenotypic modulation and is associated with vascular diseases.

Myocardial ischemia/reperfusion (I/R) injury predominantly occurs in coronary artery diseases when prompt reperfusion is implemented to salvage the ischemic myocardium. Cardiomyocyte death represents a significant contributor to this injury. Recently, a growing body of research has established that MAMs play a regulatory role in cardiomyocyte death. As previously mentioned, MAMs are crucial in governing mPTP opening induced by Ca^2+^ overload, which serves as a major contributor to cardiomyocyte death during reperfusion damage [7]. CypD, a mitochondrial matrix protein, serves as a central regulator of mPTP opening and necrosis. Elevated CypD levels are sufficient to induce mPTP activation independent of external apoptotic or necrotic stimuli [233]. The CypD–IP3Rs–GRP75–VDACs complex mainly modulates Ca^2+^ exchange in MAMs. Disruption of this complex through suppression of its components reduces mitochondrial Ca^2^⁺ overload and confers protection against hypoxia/reoxygenation (H/R) injury in cardiomyocytes [234]. Additionally, GSK‐3β, located in MAMs, interacts with the CypD‐IP3R‐GRP75‐VDAC complex, and this interaction intensifies with cell death following H/R injury [55]. The inhibition of GSK‐3β impairs IP3R function, relieves mitochondrial Ca^2+^ overload, mitigates cell death, and reduces the infarct area in reperfusion heart [235]. In addition, temporary MFN1/2 knockout may reduce mitochondrial Ca^2^⁺ overload and alleviate oxidative stress during reperfusion, offering short‐term cardioprotection, while prolonged MFN2 deletion impairs autophagy and mitochondrial fusion, leading to eventual cardiac dysfunction [236]. CL is also implicated in the progression of cardiomyocyte death. The oxidation of CL is regarded as an apoptotic signal, facilitating cytochrome *c* release from mitochondria, reducing mitochondrial membrane potential, and ultimately triggering cell death [237]. Extensive experimental and clinical evidence has demonstrated that UPR is activated during I/R injury due to diverse pathological insults. XBP1s plays a protective role in the heart during I/R injury in mice [238]. Furthermore, XBP1 mRNA undergoes splicing during I/R, potentially acting as a positive modulator of ER function in cardiac I/R injury [239, 240]. Besides the beneficial roles of MAMs in I/R injury, downstream targets of PERK, such as p53, upregulated modulator of apoptosis (PUMA) and CHOP, may contribute to cell death induced by ER stress following I/R injury [241] (Figure 6A). Additionally, ER stress signaling can interact with pathways governing other modes of cell death, thereby contributing to myocardial I/R injury. Notably, in mice subjected to acute myocardial infarction, receptor‐interacting serine/threonine‐protein kinase 3 (RIPK3), a key necroptotic mediator, triggers ER stress via a RIP3‐Ca^2^⁺‐calmodulin‐dependent protein kinase II (CAMKII) signaling axis [242]. What is more, accumulating evidence has demonstrated the significant roles of mitochondrial dynamics and mitophagy regulated by MAMs in I/R injury. Mitochondrial fragmentation during ischemia is associated with elevated cell death; however, mitochondrial fission primarily serves to produce additional mitochondria to meet cardiomyocytes’ energy demands during I/R [243]. Suppressing mitochondrial fission through DRP1 inhibition reduces the vulnerability of HL‐1 cells to mPTP opening and attenuates I/R‐induced cell death [244, 245, 246]. Furthermore, during the context of I/R, upregulation of MCU activates calpain, which in turn downregulates OPA1, culminating in mitochondrial fission [247]. The removal of aged or dysfunctional mitochondria through MAMs‐mediated mitophagy plays a critical role in sustaining myocardial health under I/R conditions. In contrast, I/R injury impairs this process and facilitates apoptosis [244, 248]. Dysregulation of the Sirt1/Sirt3 axis during myocardial I/R suppresses the PINK1/Parkin pathway, impairing mitochondrial ubiquitination and disrupting mitophagy in cardiomyocytes, which ultimately results in ferroptosis, lipid peroxidation, and ROS accumulation [249]. Conversely, activation of FUNDC1 by the AMPK–ULK1 axis can enhance mitophagy and preserve mitochondrial functions, thereby reducing ROS production, inhibiting the recruitment of NLRP3 inflammasomes, and preventing cell pyroptosis. Eventually, this leads to a reduction in myocardial fibrosis and excessive myocardial remodeling [250].

#### 4.4.2 Heart Failure

Heart failure (HF) is a multifactorial and progressive disorder initiated by cardiac dysfunction and sustained by continuous neurohormonal signaling and progressive adverse remodeling of cardiac tissue [251]. MAMs‐resident enzymes, possessing diverse functions, might be implicated in HF progression and potentially serve as future therapeutic targets. HF is characterized by necrosis associated with Ca^2+^ overload (Figure 6A). Mechanistically, the knockdown of CypD, a regulator of the mPTP underlying necrosis, inhibited Ca^2+^ influx‐induced myocyte necrosis, HF, and isoproterenol‐induced premature death [252]. Conversely, patients with HF exhibit decreased FUNDC1 expression along with impaired SR–mitochondria contacts [253, 254]. Disruption of FUNDC1–IP3R2 interactions can cause a reduction in mitochondrial Ca^2+^, potentially resulting in mitochondrial dysfunction and promoting the development of cardiac dysfunction and HF [56]. The peroxidation of CL prompts the release of cytochrome *c* into the cytoplasm, a process that activates caspase‐3 to trigger cellular apoptosis [255]. In HF, the loss of CL amplifies ROS production, which further exacerbates CL peroxidation. CL deficiency has also been detected in both animal models of HF and in explanted failed human hearts [256]. In patients with HF, cardiomyocytes exhibit heightened ER stress, accompanied by structural remodeling and altered expression of ER and UPR components [257, 258]. The levels of XBP1s, BiP, ATF4, and CHOP are markedly increased in failing hearts [259, 260]. The ER stress and UPR, finely tuned by MAMs, drive cardiac hypertrophy and HF through activation of the PERK–EIF2α–ATF4–CHOP signaling axis [261]. Additionally, prominent ER stress can be detected in both hypertrophic and failing mouse hearts [262]. ER stress signaling, particularly CHOP‐induced apoptosis, is evident in hypertrophic and failing hearts subjected to transverse aortic constriction (TAC) [263]. These findings underscore the pivotal contribution of ER stress to the development of cardiac hypertrophy and HF [263]. Another MAMs‐associated protein implicated in HF is Sig‐1R. Its inhibition enhances autophagy in cardiomyocytes under oxidative stress, whereas its activation promotes mitochondrial Ca^2^⁺ transfer and ATP production, thereby attenuating angiotensin II‐induced hypertrophy and cardiomyocyte injury [264, 265]. A growing number of studies suggest that aberrant mitochondrial fusion and division also contribute to the progression of HF as a consequence of disordered MAMs. Reduced OPA1 expression and the presence of small mitochondrial fragments are observed in failing hearts, signifying diminished mitochondrial fusion [266]. Compared with normal cells, MFN1/2‐deficient cells display impaired mitochondrial fusion and aberrant mitochondrial morphology [267]. In the left ventricular myocardium of dogs with chronic HF, MFN2, and OPA1 are significantly downregulated, while DRP1 and FIS1 are upregulated, indicating a pronounced dysregulation of fission and fusion dynamics [268]. In cardiac pathology, DRP1 and FIS1 are upregulated, and excessive mitochondrial fission adversely affects heart function in HF. In failing hearts, DRP1 hyperactivation through S616 phosphorylation promotes mitochondrial injury and contributes to cardiac dysfunction [269]. Compelling evidence shows that mitophagy‐mediated removal of dysfunctional mitochondria is essential for preserving mitochondrial integrity and cardiac function [270]. PINK1, FUNDC1 and Bcl‐2‐interacting protein 3 (BNIP3), located on the OMM, are also involved in the mitochondrial degradation mechanism [271, 272]. Defective mitophagy worsens mitochondrial dysfunction and HF in animal models, whereas enhancing cardiac mitophagy confers cardioprotective effects [273, 274, 275].

#### 4.4.3 Atherosclerosis

Atherosclerosis represents a chronic inflammatory disease. The initial event in plaque development is endothelial dysfunction [276]. VSMCs, situated in the media layer of the vessel wall, are a major contributor to the different stages of atherosclerotic plaque progression, exhibiting significant plasticity and particular clonality [277]. Uncontrolled phenotypic switching of VSMCs constitutes a crucial pathological feature of atherosclerosis [275]. Notably, mitochondrial dysfunction can drive these pathological changes in VSMCs, thereby accelerating disease progression [278, 279] (Figure 6B). The underlying mechanisms predominantly involve Ca^2+^ overload, ER stress, oxidative stress, and disrupted mitochondrial dynamics [280]. Ca^2+^ fulfills several crucial functions within mitochondria under the modulation of MAMs proteins. Specifically, it is involved in activating oxidative phosphorylation and regulating ROS production [281]. Given its properties, Ca^2+^ is also intimately related to VSMCs phenotypic transition and metabolic regulation [282]. The MCU serves as the rate‐limiting protein for Ca^2+^ uptake [283]. When excessive Ca^2+^ is transported into mitochondria via MCU, the expression of DRP1 rises, subsequently inducing VSMCs proliferation [284]. Moreover, oxidized low‐density lipoprotein (oxLDL) can augment the cytoplasmic Ca^2+^ concentration [285], activating the Ca^2+^‐dependent apoptosis in VSMCs [286]. Recently, the disruption of lipid homeostasis in atherosclerosis has attracted increasing attention. CL, an IMM phospholipid that relocates to the OMM after mitochondrial damage, is highly susceptible to peroxidation. Oxidized CL elevates intracellular Ca^2^⁺ and upregulates ICAM‐1 and VCAM‐1 in ECs [287]. ROS functions as another essential mediator, which is regulated by MAMs, in modulating the phenotypic characteristics of VSMCs. It promotes vascular remodeling by stimulating VSMCs proliferation through enhanced MAPK activity and increased DNA synthesis [288]. ER stress and maladaptive UPR contribute to atherosclerosis, as evidenced by increased BiP and CHOP levels in human coronary arteries [289]. In murine models, ER stress facilitates the development of vulnerable plaques by triggering macrophage apoptosis through UPR activation and CHOP induction in response to cholesterol overload [290]. Recently, mitochondrial dynamics has been associated with VSMCs phenotypic transformation and reported to regulate VSMCs proliferation via cell cycle modulation [291]. The imbalance of mitochondrial dynamics leads to uncontrolled VSMCs phenotypic modulation. Increased mitochondrial fission endows the synthetic phenotype with hyperproliferative features and promotes VSMCs migration through DRP1 binding to the OMM and DRP1 phosphorylation at ser616 [292]. Additionally, enrichment of DRP1 has been detected in calcified carotid arteries, while partial DRP1 deficiency in mice confers resistance to vascular calcification during atherosclerosis [293]. Downregulation of DRP1 attenuates mitochondrial fission and restrains VSMCs phenotypic conversion [294]. Furthermore, the expression of DRP1 receptors like FIS1, MiD49, and MiD51 is also elevated in pathological VSMCs [295]. In contrast, promoting mitochondrial fusion can counteract VSMCs phenotypic switching. MFN2 facilitates the formation of mitochondrial networks and ER tethering, which inhibits VSMCs proliferation [296].

#### 4.4.4 Pulmonary Hypertension

Pulmonary hypertension (PH) is a disorder defined by an increase in mean pulmonary artery pressure that arises from a variety of underlying causes [297]. The modifications of pulmonary artery smooth muscle cells (PASMCs) constitute the typical pathological characteristics of PH [298, 299]. The pyruvate dehydrogenase (PDH) complex acts as a gatekeeper in the process of glucose oxidation within mitochondria and is of critical importance in the metabolic transformation of PASMCs [300]. Ca^2^⁺ transmitted via MAMs regulate PDH phosphorylation and inhibit PDH kinase. Consequently, this enhancement of PDH activity promotes glucose oxidation [301]. Inhibiting mitochondrial oxidation decreases mitochondrial membrane hyperpolarization and ROS production, which raises the threshold for mPTP opening and delays apoptosis in PASMCs [300] (Figure 6B). Upon membrane potential loss, PINK1 accumulates on the OMM, recruiting the E3 ligase Parkin from the cytosol. Parkin then ubiquitinates OMM proteins, triggering the clearance of damaged mitochondria [302]. Mitochondrial dynamics are closely linked to phenotypic changes in PASMCs. Notably, PASMCs derived from PH patients show fragmented mitochondria, with increased level of DRP1 and decreased level of MFN2. Epigenetic upregulation of DRP1 adaptors, MiD49 and MiD51, drives mitotic fission, fostering apoptosis resistance and abnormal cellular proliferation in PH [293]. Excessive activation of mitochondrial fusion represents another form of mitochondrial imbalance, resulting in diminished PASMCs proliferation and enhanced apoptotic activity. Such outcomes are observed in mice with MFN2 overexpression, whereas MFN2‐deficient mice show elevated PASMCs proliferation [303]. Analogously, MFN1 is also associated with excessive proliferation of PASMCs [304]. PASMCs are also the locus where excessive mitophagy can transpire. Under hypoxic conditions in PH, FUNDC1‐dependent mitophagy is enhanced, which activates the ROS–HIF1α axis and drives PASMCs proliferation, ultimately facilitating pulmonary vascular remodeling. FUNDC1 knockdown, however, attenuates mitophagy and inhibits the proliferation of PASMCs [305, 306, 307, 308].

Actually, MAMs are also associated with many other cardiovascular diseases. Recent studies have suggested that there is a strong link between MAMs and the progression of cardiomyopathy, especially diabetic cardiomyopathy (DCM) [309]. High glucose‐induced disruption of MAMs integrity and mitochondrial dysfunction have been implicated in the development of DCM [309]. A previous study demonstrated that enhanced glucose levels led to upregulation of FUNDC1, IP3R2, and enhanced MAMs formation, which in turn contributed to increased Ca^2+^ transfer, resulting in mitochondrial dysfunction [254]. Furthermore, an in vitro study revealed that cardiomyocytes exposed to high glucose conditions exhibited excessive mitochondrial fission accompanied by reduced MFN2 expression [310]. Additionally, inhibition of PERK was linked with reduced cardiomyocyte apoptosis in high glucose environments, primarily through the suppression of ROS‐induced PERK pathway activation that triggers ER stress‐mediated apoptosis [311].

#### 4.4.5 Crosstalk of MAMs‐Regulated Cellular Processes in CVDs

MAMs are associated with a diverse array of intercellular processes as previously mentioned. These regulated events not only function independently in the progression of CVDs, but also exhibit synergistic effects due to their inherent crosstalk. For instance, the excessive accumulation of Ca^2+^ in the cytosol triggers various signaling mechanisms, thereby inducing apoptosis via mPTP opening and initiating mitophagy [312]. ER stress can also be activated by Ca^2+^ overload, accompanied by mPTP opening and enhanced ROS generation mediated by RIP3 [313]. Notably, elevated ROS levels can drive a shift in mitochondrial dynamics from fusion to fission. Conversely, disruption of these dynamics can, in turn, promote excessive ROS production [295]. Detrimental stimuli can activate VSMCs’ mitochondrial fission, concurrent with elevated mitochondrial ROS levels [314]. Additionally, ischemia induces mitochondrial fragmentation, which predominantly depends on DRP1 and is associated with Ca^2+^ overload and increased ROS release. The classical mitochondrial lipid, CL, is also intertwined with these processes. In HF mouse model, the depletion of CL amplifies ROS production, which further exacerbates CL peroxidation and establishes a vicious cycle, resulting in additional mitochondrial dysfunction and ultimately cardiomyocyte death [315]. The accumulation of oxidized CL in the OMM also leads to mPTP opening and the release of cytochrome *c*, thereby triggering programmed cell death [112].

### 4.5 Common Regulatory Mechanisms of MAMs Across the Diseases

Through the description of the above phenomena, we can observe that some classical pathways play regulatory roles in the progression of various diseases. For instance, dysregulation of IP3R‐mediated Ca^2^⁺ flux induces cell death across multiple cell types, including cancer cells, neurons, pancreatic β cells, and heart cells. GSK3β, through its interaction with the IP3Rs–GRP75–VDACs complex, enhances a tissue‐specific ER–mitochondrial Ca^2+^ leak in murine pancreatic β cells and intensifies cell death in H9c2 cardiomyoblasts [316]. The IRE1α–XBP1 UPR branch is also implicated in different diseases. For instance, IRE1α–XBP1 axis promotes angiogenesis and cell proliferation in breast cancers and enhances metabolic inflammation and hepatocyte proliferation in hepatocellular carcinoma [177, 178]. XBP1s has protective roles in I/R injury in mice heart [238]. PINK1/Parkin‐mediated mitophagy serves as defender of mitochondria and helps maintaining cellular homeostasis. In the early stage of a long‐term HFD‐induced mouse model of NAFLD, mitophagy is upregulated as a compensatory mechanism to counteract hepatic lipid accumulation [230]. Similarly, Parkin‐mediated mitophagy is enhanced in mutant hAPP neurons and in the brains of AD patients [202]. As for adverse cardiac mitochondrial function in HF, mitophagy, linked with ventricular dysfunction and pathological cardiac hypertrophy, is downregulated for the loss of PINK1 [271].

### 4.6 Interaction Between MAMs and the Pathological Microenvironment

Most of the diseases discussed above have been shown to be associated with disruptions in systemic homeostasis, especially cancers, metabolic diseases and cardiovascular diseases. Therefore, exploring the dysregulation of MAMs in relation to changes in the pathological microenvironment is both necessary and of potential therapeutic value. Hypoxia in nasopharyngeal carcinoma is associated with reduced expression of MFN1 and MFN2, key mediators of mitochondrial fusion, thereby promoting a shift toward smaller and fragmented mitochondrial phenotypes [317]. This process may be mediated by a hypoxia‐induced signaling cascade involving the miR‐24–Myc–MFN1 axis, which ultimately leads to reduced MFN1 expression [317]. Hypoxia within the tumor microenvironment activates the mTOR–DRP1 signaling axis in NK cells, thereby driving excessive mitochondrial fission [318]. Diabetes is characterized by impaired glucose homeostasis. MAMs serve as critical platforms that integrate cellular energy sensing with mitochondrial function, thereby contributing to the regulation of glucose balance [217]. In hepatocytes, IP3R knockdown decreases glucose production [319]. And the accumulation of lipid leads to aberrant mitophagy. Changes in redox status also have a significant impact on the cardiovascular diseases. Both hypoxia and oxidative stress can contribute to pathological progression. ROS‐induced oxidation of CL is regarded as an apoptotic signal, which promotes mitochondrial cytochrome *c* release into the cytosol, activates the apoptotic cascade, and ultimately intensifies I/R injury [237]. While in hypoxic PH, FUNDC1‐mediated mitophagy is upregulated and stimulates PASMCs proliferation, leading to pathological proliferation in PH. To sum up, alterations in the pathological microenvironment can lead to MAMs dysfunction, which in turn further exacerbates the pathological microenvironment, forming a bidirectional vicious cycle.

## 5 Pharmacological Modulation Targeting MAMs in Diseases

The characteristic functions of MAMs, such as their involvement in Ca^2^⁺ transfer, lipid metabolism, ER stress, and oxidative stress, as well as mitochondrial dynamics and mitophagy, endow them with the potential to serve as diagnostic markers and therapeutic targets for common diseases. In this section, a comprehensive overview of the extant synthetic medications and clinical trials which are directed toward MAMs within the framework of common diseases will be provided.

### 5.1 Ca^2^⁺ Transfer‐Targeting Agents

GSK3β inhibitors: GSK3β, situated at MAMs, physically interacts with the CypD–GRP75–IP3R–VDAC axis, and this interaction intensifies after H/R injury, coinciding with elevated levels of cell death [55]. The inhibition of GSK3β can reduce IP3R activity, thereby restricting the cytosolic and mitochondrial Ca^2^⁺ overload, which subsequently leads to a decrease in cell death and infarct size in reperfused regions.

Fluvoxamine: Sig‐1R can enhance mitochondrial Ca^2^⁺ transport and ATP production, consequently inhibiting angiotensin II‐induced myocardial hypertrophy [320]. Fluvoxamine binds strongly to Sig‐1R and remarkably ameliorates HF and cardiac dysfunction in TAC models of both mice and rats [321].

### 5.2 Lipid Metabolism‐Regulating Agents

1. Dafaglitapin (Dafa): Myocardial infarction strongly elevates the expression of acyl‐coenzyme A: lysocardiolipin acyltransferase‐1 (ALCAT1), connecting hypoxic injury with oxidative stress, loss of tetralinoleoyl cardiolipin (TLCL), and impaired mitochondrial function. Dafa effectively and specifically targets ALCAT1, restoring TLCL levels and mitochondrial respiration via downregulation of HIF1α‐driven pathways [322].
2. MG53: Under oxidative stress, MG53 binds to cardiolipin in a dose‐dependent manner to maintain mitochondria integrity [323].
3. PD‐132301: PD‐132301 is an inhibitor of sterol O‐acyltransferase 1 (SOAT1; also known as ACAT1). Radiolabeled ACAT1 inhibitors can facilitate drug discovery and assist in the diagnosis of ACAT1‐related disorders, such as atherosclerosis [324].
4. Avasimibe: Avasimibe can effectively inhibit ACAT1 and, thus, has been employed in the treatment of atherosclerosis [325].

### 5.3 Agents Modulating ER Stress and Oxidative Stress

1. Statins: Statins possess a multitude of pharmacological effects that are associated with the attenuation of ER stress and oxidative stress [326]. Notably, atorvastatin exerts a protective role in the myocardium against I/R injury and the development of HF following myocardial infarction. This may occur by reducing apoptosis resulting from CHOP expression, JNK phosphorylation, and caspase 12 activation [327].
2. 2‐APQC: 2‐APQC is a novel small molecule activator of SIRT3, which helps regulate cardiac fibrosis and HF. The SIRT3‐pyrroline‐5‐carboxylate reductase 1 (PYCR1) axis is closely related to HF. By activating PYCR1, 2‐APQC enhances mitochondrial metabolism and inhibit the ROS‐p38 mitogen‐activated protein kinase (p38MAPK) pathway, protecting against isoproterenol‐induced mitochondrial oxidative damage [328].
3. Bu–Shen–Tian–Jing Formula (BSTJF): In rat models, BSTJF upregulated SIRT3, suppressed p38 MAPK activation, enhanced PI3K/AKT signaling, and reduced oxidative stress. In KGN cells, BSTJF restored impaired glucose uptake and SIRT3 levels, while also decreasing mitochondrial ROS production [329].

### 5.4 Mitochondrial Manipulation‐Targeting Agents

1. Omentin1: Omentin‐1, a recently identified adipokine, has protective effects in myocardial ischemia‐induced HF [330]. In HF mice, omentin‐1 promotes mitochondrial fusion and reduces fission, associated with increased OPA1 and MFN2 levels and decreased phosphorylation level of DRP1 at serine 616 [330].
2. Melatonin: Melatonin helps to restore mitochondrial fusion through OPA1 by activating the AMPK pathway. This maintains mitochondrial function, prevents excessive mitochondrial division, and inhibits mitochondrial apoptosis in cardiomyocytes [331].
3. Mitochondrial division inhibitor 1 (Mdivi‐1): Mdivi‐1 is a highly effective mitochondrial fission inhibitor that attenuates mitochondrial division by selectively inhibiting the mitochondrial division dynamin [332]. Mdivi‐1 treatment restored mitochondrial dynamics, reducing extracellular amyloid deposition and the Aβ1‐42/Aβ1‐40 ratio, and protected AD mice from cognitive impairment in the Y‐maze test [333].
4. Punicalagin: Punicalagin protects against diabetic liver injury by boosting mitophagy and antioxidant enzyme activity, thereby lowering oxidative stress [334].
5. Paeonol: Under hyperglycemic conditions, paeonol enhances OPA1‐dependent mitochondrial fusion, thereby mitigating oxidative stress and maintaining mitochondrial respiratory function [335].
6. UMI‐77: UMI‐77 can induce mitophagy in vivo and effectively reverse molecular and behavioral phenotypes in the APP/PS1 mice model of AD [336].
7. Exenatide: Exenatide inhibits mitochondrial fission by promoting the phosphorylation of DRP1 at serine 637, thereby impairing DRP1 translocation to the mitochondria [337]. Exenatide can contribute to the amelioration of HF [338].

### 5.5 Clinical and Preclinical Trials of MAMs‐Targeting Drugs

With continued investment, more drugs are being discovered with ongoing research. Yet studies on MAMs modulators are still limited, with most evidence sourced from the clinical and preclinical studies summarized in Table 2.

**TABLE 2:** Clinical and pre‐clinical trials targeting MAMs components

Pharmaceutical	Targeted diseases	Regulatory mechanism	Trial identifier
Rycals	Heart failure	Rycals can stabilize RYR2 channels located at MAMs, and prevent Ca^2+^ leakage from the sarcoplasmic reticulum [339].	NCT04141670
Bevacizumab	Breast cancer	Bevacizumab impairs mitochondria in ovarian cancer cells through altered mitochondrial dynamics, increased mitophagy, and apoptosis [340].	NCT02806817
Metformin	Diabetes	Metformin supports mitochondrial function, maintains ETC complex levels, and boosts mitophagy in the treatment of diabetes [341].	NCT01813929
Sulforaphane	Heart failure	SFN inhibits the expressions of acyl‐coenzyme A: diacylglycerol acyltransferases 2 (DGAT2) and ACAT1, the key enzymes responsible for triacylglyceride and cholesterol ester synthesis [342].	NCT05408559
Berberine	Non‐alcoholic fatty liver	Berberine has a protective role in preventing vascular endothelial cell damage and delaying the progression of atherosclerosis by reducing the stability of ACSL4 to counteract ferroptosis caused by lipid peroxidation [343].	NCT05523024
Punicalagin	Colorectal cancer	Punicalagin stimulates OPA1‐driven mitochondrial fusion through inhibition of PTP1B, which enhances STAT3 phosphorylation and upregulates Opa1 expression, ultimately conferring protection in DCM [344].	NCT01916239
Irisin	Myocardial infarction	Irisin activated AMPK and downregulated the expression of DRP1, alleviating the mitochondrial fission and VSMCs osteoblastic transformation [294].	NCT02498431
Quercetin	Coronary artery disease	Quercetin, a natural flavonoid, activates mitophagy and enhances ROS clearance in cardiomyocytes during H/R, thereby providing protective effects [345].	NCT04907253
Pridopidine	Huntington's disease	The Sig1R is a molecular chaperone regulating several cellular pathways in neurons and glial cells. Pridopidine is a highly selective Sig1R agonist and shows efficacy in neurodegenerative diseases [346].	NCT01306929
Spermidine	Myocardial infarction	Spermidine promotes PINK1‐mediated mitophagy, thereby improving mitochondrial integrity [347].	NCT06186102

## 6 Perspectives

Given the role of MAMs dysfunction in various diseases, the study of MAMs has become an increasingly dynamic field. Recently, with the emergence of more advanced technologies for investigating MAMs, we have gained a deeper understanding of MAMs. However, MAMs possess complex structures and functions, and will react distinctively depend on metabolic states and stress conditions. Debates on the cell‐type‐dependent roles of MAMs in regulating cellular processes, limitation of effective targeted drugs and controversial topics regarding the regulatory mechanisms of MAMs still exist and need to be resolved.

### 6.1 Cutting‐Edge Technologies of Studying MAMs

MAMs were first discovered and described in 1958 with the aid of transmission electron microscope. In the 1990s, Vance first separated and characterized the MAMs fraction. Then, in 2009, a detailed protocol for isolating MAMs was established and published in *Nature* Protocols [5]. With technological advances, an increasing number of novel techniques have been applied to directly visualize the presence and structure of MAMs (Figure 1B). The ER and mitochondria are labeled with red and green fluorescent markers respectively and can be detected through immunofluorescence [28]. Cryoelectron microscopy and three‐dimensional electron microscopy with high‐speed molecular tracking of a model organelle tether are also used to visualize and reconstruct the 3D architecture of MAMs [27, 29]. MAMs‐spGFP is also used to detect the formation of MAMs. A green fluorescent signal is generated upon close apposition of the ER and OMM [348]. In addition to the direct visualization of MAMs’ structure, a variety of functional assays have also been developed to assess the functional pathways modulated by MAMs. The control of Ca^2^⁺ signaling by MAMs is essential for a variety of cellular functions. MAMs‐Calflux, a newly developed BRET‐based probe, enables precise monitoring of Ca^2^⁺ at MAMs, allowing visualization of its heterogeneous intracellular distribution and detection of abnormal accumulation [349]. To enhance MAMs‐regulated signaling pathways, several tools have also been developed. For example, an adenovirus expressing a synthetic ER–mitochondria tethering protein can be used to enhance the MAMs’ tethering [350]. The availability of this tool can be validated by using TEM and confocal live‐cell imaging [350].

### 6.2 Cell‐Type‐Dependent Roles of MAMs in Regulating Cellular Processes

MAMs are a subcellular structure that exhibits a largely conserved composition among different cell types. However, in different cell types, MAMs may exert distinct regulatory effects through modulation of the same signaling pathway. For example, the IP3Rs–GRP75–VDACs complex plays a central role in controlling Ca^2^⁺ transfer from the ER to mitochondria, maintaining optimal Ca^2^⁺ concentrations to support TCA cycle and ETC function, thereby boosting ATP generation, and regulating metabolic processes by affecting glucose transporter activity [67, 351]. However, as a double‐edged sword, Ca^2+^ overload can trigger distinct cell fate across different cell types, some of which may have harmful effects on the human body. In most cancer cells, enhanced Ca^2+^ increases the sensitivity to apoptotic stimuli and plays a protective role in the progression of cancers [352]. While in cardiomyocytes, excessive Ca^2+^ is a vital mediator of cardiomyocyte death during I/R injury [7]. The opening of mPTP triggered by Ca^2+^ overload represents a strong proapoptotic signal [233]. This phenomenon is also observed in all the other cardiac cell types including endothelial cells, fibroblasts, and vascular smooth muscle cells. Mitochondrial fission also plays distinct roles in different cell types. In neurons, reduced mitochondrial fragmentation decreases their presence in dendritic spines and hinders synaptic development, while enhancing mitochondrial fission promotes synapse formation [353]. On the contrary, in the pancreatic cells, increased mitochondrial fission leads to the induction of normal cell transformation into cancer cells [181]. The increased mitochondrial fission also results in further metastasis in hepatocellular carcinoma [182]. In cardiomyocytes, mitochondrial fission under ischemic conditions also leads to adverse events, and is correlated with increased cell death [354]. These observations collectively demonstrated that MAMs can exert markedly different regulatory effects across distinct cell types by modulating the same signaling pathway. It also creates a gap in in translating mechanistic studies of MAMs into effective targeted therapies. It is often difficult to achieve therapeutic effects by specifically targeting a MAMs‐regulated signaling pathway without causing side effects. Thus, it is crucial to develop strategies for selectively targeting MAMs in specific cell types to achieve improved therapeutic outcomes. Nevertheless, research in this area is still in early stage, and the majority of evidence comes from clinical and preclinical studies.

### 6.3 Discussion on Existing Contradictions and Disputes

In addition, the specific mechanisms through which MAMs exert regulatory effects on cell fates are also required further in‐depth exploration, with the anticipation that more effective therapeutic strategies could thereby be established. For instance, traditionally, mitochondrial fission is viewed as vital for quality control by removing damaged mitochondria and, under severe stress, promoting both mitophagy and apoptosis [354]. Recently, Kleele et al. identified two mechanistically distinct forms of mitochondrial fission. Peripheral division facilitates the removal of damaged components by generating smaller mitochondria destined for mitophagy, whereas midzone division supports mitochondrial proliferation [355]. Different fission mechanisms result in disparate mitochondrial fates. In contrast to previous research suggesting that fission underlies mitochondrial degradation, the positioning of the fission site might be a crucial morphological determinant of the decision to proliferate or degrade. This phenomenon has implications in pathological contexts. If only one type of fission is dysregulated under pathological conditions, the use of global fission inhibitors might further disrupt cell homeostasis. This indicates that MAMs might possess distinct regulatory functions in the same intracellular process, and thus more rational and specific therapeutic targets are urgently required in the future.

### 6.4 MAMs as Integrative Therapeutic Targets: Future Directions

MAMs function as critical regulatory hubs integrating multiple cellular processes, including Ca^2+^ transfer, lipid metabolism, mitochondrial dynamics, and ER stress response. Targeting MAMs offers the unique advantage of modulating several interconnected pathways simultaneously, thereby enabling a more comprehensive therapeutic effect. Moreover, their central role in maintaining cellular homeostasis under both physiological and pathological conditions makes MAMs attractive candidates for interventions aimed at complicated diseases such as cancers, neurodegeneration, metabolic disorders, and cardiovascular diseases [356]. Accumulating evidence positions MAMs as a fertile landscape for the discovery of novel therapeutic targets, and the strategic modulation of MAMs‐associated signaling pathways holds great promise for the development of next generation therapies. However, despite their promise, several challenges also exist and hinder the translation of MAMs‐targeted therapies into clinical practice. First, the structural and functional complexity of MAMs complicates precise target identification. It is challenging for drugs to precisely target proteins regulating specific signaling pathways. Second, the cell type‐ and context‐specific nature of MAMs regulation demands highly selective strategies to avoid unintended disruption of physiological functions [2]. However, given the involvement of MAMs in multiple intercellular processes, there exist the risk of off‐target effects, requiring the balance of therapeutic efficacy and safety. Finally, efficient and specific delivery of therapeutics to MAMs‐associated proteins remains technically challenging. The development of targeted delivery systems, such as nanoparticle‐based carriers, peptide ligands, or organelle‐specific drug conjugates, may overcome current barriers in specificity and reduce off‐target toxicity [357].

## 7 Conclusions

Over the past decades, MCSs—particularly MAMs—have gained extensive attention. Recent years, concomitant with the evolution of measurement modalities, have witnessed significant progress in deciphering the architecture and diverse functions of MAMs including Ca^2+^ translocation, lipid metabolism, ER stress, oxidative stress, mitochondrial dynamics, and mitophagy [26]. Essentially, MAMs constitute a pivotal microdomain, acting as an interface for ER–mitochondria crosstalk and expediting the rapid exchange of biomolecules requisite for cellular homeostasis. As a core mediator of a plurality of cellular processes, aberrant MAMs participate diverse pathophysiological processes via elaborate signaling cascades. The past two decades have been characterized by intensive exploration of MAMs‐regulated mitochondrial and ER‐correlated molecular machineries in health and diseases. Based on previous studies, researchers have deduced that MAMs contribute to the progression of various diseases and are becoming promising targets in pharmaceutical research and development. Unraveling the regulatory mechanisms underpinning MAMs integrity may facilitate the discovery of novel therapeutic strategies against diseases.

In this review, we have systematically summarized the structure of MAMs as well as their regulatory functions and underlying mechanisms in healthy state, and have illustrated them with detailed and visually appealing figures. Meanwhile, we have conducted a detailed analysis of the association between MAMs and all related diseases, which are complex and comprehensive. We have also detailedly summarized molecule compounds targeting MAMs, along with relevant clinical studies. In the end, we introduced the latest technological advances related to MAMs, discussed the cell‐type‐dependent roles of MAMs in regulating cellular processes, and explored several controversial topics in this field. Though as a structure with complex functions, the exploration of MAMs is far from completed. We believe that targeting MAMs will be a promising treatment for diseases in the future with the aid of ongoing scientific progress.

## Author Contributions

Hua Li and Junbo Ge proposed the conception and study design and had the final approval of the manuscript submitted. Hangnan Hong and Zhenyang Guo participated in the data collection and analysis, the drafting of the manuscript and the submission. All the authors have read and approved the final manuscript.

## Ethics Statement

The authors have nothing to report.

## Conflicts of Interest

The authors declare no conflicts of interest.

## Data Availability

The authors have nothing to report.
